# Multi-Omics Identification of Vasculogenic Mimicry-Associated Molecular Subtypes in Hepatocellular Carcinoma for Prognostic Stratification and Therapeutic Response Prediction

**DOI:** 10.3390/cancers18152482

**Published:** 2026-08-02

**Authors:** Yuting Tao, Shuzhen Liao, Tao Liu, Ruyi Lai, Chao Feng, Qiuyan Wang

**Affiliations:** 1 Institute of Life Sciences, Guangxi Medical University, Nanning 530021, China; taoyuting@gxmu.edu.cn (Y.T.); ry6798@126.com (R.L.); 2 Guangxi Key Laboratory for Genomic and Personalized Medicine, Guangxi Medical University, Nanning 530021, China; l1utao@sr.gxmu.edu.cn; 3 Center for Genomic and Personalized Medicine, Guangxi Medical University, Nanning 530021, China; 4 The Academician Workstation on Metabolism and Health, The First Affiliated Hospital of Guangxi Medical University, Nanning 530021, China; 5 School of Information and Management, Guangxi Medical University, Nanning 530021, China; 202421222@sr.gxmu.edu.cn

**Keywords:** vasculogenic mimicry, hepatocellular carcinoma, molecular subtyping, multi-omics

## Abstract

**Objective:** Vasculogenic mimicry (VM), characterized by the de novo formation of microvascular-like channels derived from aggressive tumor cells without involving traditional endothelial cells, is a pivotal pathological hallmark driving extreme invasiveness and dismal prognosis in hepatocellular carcinoma (HCC). This study aimed to establish a VM-based molecular subtyping system and systematically characterize its associated biological features, thereby providing a potential framework for individualized prognostic assessment and treatment decision-making in HCC. **Methods:** We integrated curated VM-associated gene sets with single-cell RNA sequencing data to identify malignant epithelial cell-enriched VM-associated candidate genes. Subsequently, univariate Cox regression, LASSO-Cox regression, and multivariate Cox regression were sequentially performed to identify six prognostic VM-related genes: *HSPA9*, *TGFA*, *MAD2L1*, *PROM1*, *AGXT*, and *GCGR*. HCC patients were stratified into VM, Mixed-VM, and Non-VM subtypes according to VM scores. Kaplan–Meier survival analysis, time-dependent ROC analysis, and Cox regression were used to assess prognostic performance. The biological features of the classification system were evaluated using bulk transcriptomic cohorts, spatial transcriptomics, Cytometry by Time-of-Flight (CyTOF), metabolomics, lipidomics, somatic mutation and copy number alteration analyses, and treatment-related HCC cohorts. **Results:** The VM score-based classification stratified HCC patients into three molecular subtypes with distinct prognostic and biological characteristics. Patients classified as the VM subtype had significantly poorer overall survival than those classified as Mixed-VM or Non-VM subtypes, and this prognostic pattern was validated across independent cohorts. Multi-omics analyses showed that the VM subtype was associated with YAP-TAZ-TEAD-related transcriptional programs, stemness/proliferation-related features, immunoregulatory and exhaustion-like tumor microenvironmental characteristics, and distinct metabolic and lipidomic alterations involving modified nucleosides, keto acid-related metabolites, cholesteryl esters, and sphingolipid-related species. Spatial transcriptomics revealed focal enrichment of VM-score-high regions and their association with YAP-TAZ-TEAD and immune checkpoint-related signatures. In orthotopic HCC mouse models, YAP1 overexpression increased PAS^+^/CD34^−^ VM-like structures, whereas verteporfin treatment reduced these structures. In two treatment-related cohorts, the Non-VM subtype showed higher response rates to sorafenib and transarterial chemoembolization (TACE) than the VM subtype. Connectivity Map (CMap)-based computational drug prioritization and molecular docking analysis prioritized ivermectin as a candidate compound; however, its antitumor activity requires further experimental validation. **Conclusions:** This study establishes a VM score-based molecular classification framework for HCC and identifies VM-subtype-associated prognostic, spatial, immune, metabolic, genomic, and therapeutic features. These findings provide a candidate framework for molecular risk stratification and subtype-guided therapeutic exploration in HCC.

## Simple Summary

Hepatocellular carcinoma is an aggressive liver cancer that remains difficult to treat, partly because tumor cells can form their own blood vessel-like networks through a process called vasculogenic mimicry. This ability makes the cancer more invasive and less responsive to standard therapies. In this study, we developed a new classification system based on genes related to vasculogenic mimicry. Using this system, we separated liver cancer patients into three groups with distinct biological behaviors and treatment outcomes. We found that one group, with the highest vasculogenic mimicry activity, had worse survival and showed unique features in their genes, metabolism, and immune environment. Importantly, this group responded poorly to common treatments like sorafenib, but our analysis identified a potential existing drug, ivermectin, that might target these aggressive tumors. This classification system offers a practical tool to predict patient outcomes and could guide more personalized treatment decisions in the future.

## 1. Introduction

Liver cancer is a major global health burden, ranking sixth in incidence and third in cancer-related mortality worldwide, with a particularly high prevalence in China due to the country’s unique spectrum of hepatitis-related liver diseases [1]. Although surgical resection and local ablation are effective for early HCC, postoperative recurrence remains a major obstacle to improving clinical outcomes [2]. For advanced HCC, despite the emergence of targeted therapy and immune checkpoint inhibitors, overall responses are limited and survival benefits remain modest [3,4]. These challenges are largely driven by intratumoral heterogeneity, which cannot be fully captured by current clinical staging systems such as TNM [2]. Therefore, developing novel molecular subtyping methods that reflect tumor biology and predict treatment response is critical for advancing precision medicine in HCC.

Molecular subtyping has become essential for understanding tumor heterogeneity and guiding personalized therapy in many solid tumors [5]. It enables the transition from morphological classification to functional stratification by identifying core molecular alterations and signaling networks driving tumor progression [6,7,8]. In HCC, identifying key biological drivers of aggressive phenotypes and constructing robust subtyping models is essential to dissect its complex heterogeneity [9].

Vasculogenic mimicry (VM) is an endothelium-independent vascularization mechanism in which highly invasive tumor cells form functional, vessel-like structures to support tumor growth [10,11]. In HCC, VM is strongly associated with poor prognosis, metastasis, and aggressive pathological features [12]. Importantly, VM may confer resistance to conventional anti-angiogenic therapies, limiting the efficacy of agents such as lenvatinib. While VM has been widely reported in aggressive cancers, its molecular regulation, metabolic reprogramming, and immune microenvironment interactions in HCC remain poorly understood. Moreover, there is currently no clinically validated VM-based classification system for prognostic stratification or treatment guidance.

Given these gaps, this study aimed to develop and validate a robust molecular classification system based on VM-related molecular features in HCC. Using multi-omics data from clinical samples, we systematically characterized the genomic, metabolic, and immune landscapes of VM-driven HCC subtypes. We further explored the crosstalk between VM-associated tumor cell networks and neuroendocrine-related signaling pathways. This work provides a reliable framework for prognostic stratification and personalized therapy in HCC, offering new insights to overcome treatment resistance and improve patient survival.

## 2. Methods

### 2.1. Data Sources and Cohort Information

This study aimed to construct and validate a VM-associated molecular classification model for hepatocellular carcinoma. Training data for the model were derived from tumor tissue samples of 116 patients pathologically diagnosed with HCC, collected between 1 April 2018 and 30 July 2019 from the Department of Hepatobiliary Surgery, Affiliated Cancer Hospital of Guangxi Medical University. The study was approved by the Hospital Medical Ethics Committee, and written informed consent was obtained from all enrolled patients prior to surgery.

To evaluate the generalizability and robustness of the model, independent validation datasets were acquired from multiple public databases. Hepatocellular carcinoma RNA-seq data and corresponding clinical information were downloaded from the TCGA database (TCGA-LIHC, *n* = 371). From the GEO database, we obtained two HCC transcriptome datasets (GSE10141, *n* = 80; GSE14520, *n* = 221), one HCC single-cell RNA-seq dataset (GSE149614, *n* = 10), and two treatment-related HCC transcriptome datasets with complete treatment response data: a sorafenib-treated cohort (GSE109211, *n* = 67) and a TACE-treated cohort (GSE104580, *n* = 147). In addition, an HCC spatial transcriptome dataset (HRA000437) was acquired from the GSA-Human database. For the bulk transcriptomic cohorts, FPKM expression values were used for the Guangxi training cohort and the TCGA-LIHC cohort, whereas normalized expression matrices provided by the original data submitters were used for the GEO cohorts.

### 2.2. Single-Cell RNA-seq Dataset Analysis

Single-cell RNA-seq data from 10 HCC patients in the GSE149614 dataset were analyzed using the Seurat package. After quality control, genes expressed in at least 3 cells were retained, and cells with 200–6000 detected genes and a mitochondrial gene percentage <15% were included for downstream analysis. The expression matrix was normalized using the LogNormalize method followed by identification of highly variable genes using the vst method. Principal component analysis (PCA) was firstly performed to reduce data dimensionality. Subsequently, the Harmony algorithm [13] was used to eliminate inter-sample batch effects based on PCA results for data integration. A shared nearest-neighbor graph was constructed based on the top 30 principal components after batch correction. Unsupervised clustering identified 15 cell clusters, which were annotated according to canonical marker genes as malignant cells, endothelial cells, cancer-associated fibroblasts, myeloid cells, T/NK cells, and B cells.

### 2.3. Construction and Validation of the VM-Related Prognostic Risk Model

To identify HCC-specific VM-related genes, we integrated single-cell transcriptomic data with curated VM-associated gene sets. VM-related candidate genes were first collected from the GeneCards database and the published literature, yielding 1489 VM-associated genes (Supplementary Table S1). Based on the single-cell RNA-seq dataset, malignant cells and endothelial cells were identified, and differentially expressed genes upregulated in malignant cells were screened using the criteria of log_2_FC > 0.25 and *p* < 0.05. A total of 5329 malignant cell-upregulated genes were obtained. Intersecting these genes with the 1489 VM-associated genes identified 351 HCC-specific VM-related candidate genes.

These 351 candidate genes were then subjected to prognostic model construction in the Guangxi HCC cohort. Univariate Cox regression analysis was first performed to screen survival-associated genes, followed by least absolute shrinkage and selection operator Cox regression analysis and multivariate Cox regression analysis for further model optimization. Finally, six core genes, including *HSPA9*, *TGFA*, *MAD2L1*, *PROM1*, *AGXT*, and *GCGR*, were retained to construct the VM-associated prognostic signature. The VM score for each patient was calculated using the formula: $VM\;score=-0.0622\times E_{HSPA9}-0.1683\times E_{TGFA}+0.0522\times E_{MAD2L1}+0.0176\times E_{PROM1}-0.0043\times E_{AGXT}-0.0227\times E_{GCGR}$, where $E_{gene}$ represents the normalized expression level of the corresponding gene in each patient.

Patients were classified into three molecular subtypes according to the distribution of VM scores: patients in the top 25% were defined as the VM subtype, those in the bottom 25% were defined as the Non-VM subtype, and the remaining patients were classified as the Mixed-VM subtype. Time-dependent receiver operating characteristic (ROC) curves were used to evaluate the predictive performance of the VM score for 1-, 2-, and 3-year overall survival in the training cohort. Kaplan–Meier survival analysis with the log-rank test was performed to compare survival differences among the three subtypes. In the TCGA-LIHC, GSE14520, and GSE10141 validation cohorts, VM scores were calculated using the same six genes and fixed regression coefficients derived from the Guangxi training cohort, without model refitting. The 25th and 75th percentile cutoffs were recalculated separately within each cohort according to its own VM score distribution. The prognostic value of the model was further validated in these independent external HCC cohorts.

### 2.4. Weighted Gene Co-Expression Network Analysis (WGCNA)

To explore key gene modules associated with VM subtypes in hepatocellular carcinoma, WGCNA was performed to construct a co-expression network based on the normalized bulk RNA-seq expression matrix of 58 VM and Non-VM subtype samples. First, genes were ranked by expression variability using the median absolute deviation (MAD), and the top 5000 genes with the highest expression variability were selected for subsequent analysis. Four outlier samples were excluded via sample clustering, and 54 samples were finally included for analysis. The optimal soft-thresholding power β = 6 was determined by soft threshold screening, satisfying the criteria for scale-free network construction. Accordingly, pairwise correlation coefficients between genes were converted into a weighted adjacency matrix, and the topological overlap matrix [14] was calculated. Hierarchical clustering was performed based on topological overlap dissimilarity, and genes were divided into co-expression modules using the dynamic tree cut method with a minimum module size of 30 and a merge cut height of 0.25, yielding 22 independent modules. Modules significantly associated with VM subtypes were screened by calculating the correlation between module eigengenes and clinical parameters. Among them, the blue module showed the strongest correlation with the VM subtype (r = 0.74, *p* = 2 × 10^−10^) and contained 834 genes, which were identified as the key module for subsequent functional and interaction analyses.

### 2.5. Spatial Transcriptome Data Processing and Analysis

Spatial transcriptomic data were organized and processed using the R package SpatialExperiment. Spots expressing fewer than 100 genes were excluded. To evaluate the spatial distribution of VM-related features, enrichment scores of the VM core gene set and other functional gene sets were calculated for each spot using the GeneSetScore function in SpaCET. Spatial distribution maps were then generated using the spatialFeature function to visualize the regional distribution of VM scores, functional pathway activities, and microenvironmental components in HCC tissue sections.

### 2.6. Metabolomics and Lipidomics Data Analysis

A total of 22 hepatocellular carcinoma tissue samples were subjected to untargeted metabolomic and lipidomic profiling, including 12 VM subtype samples and 10 Non-VM subtype samples, with 134 metabolites and 555 lipid species identified, respectively. Raw data were preprocessed and normalized using the MetaboAnalyst 6.0 platform [15]. Partial least squares discriminant analysis (PLS-DA) was first performed to construct a classification model, and key differential molecules were screened based on variable importance in projection (VIP) scores. Heatmaps were generated to systematically illustrate core metabolic and lipid differences between the two subtypes. Furthermore, box plots were used to visually display the differential distribution of metabolites and lipids significantly upregulated in the VM subtype between the two groups. To evaluate the subtype-discriminating ability of these molecules, receiver operating characteristic curves were plotted for major candidate metabolites and lipids, and the area under the curve was calculated to quantify their discriminatory performance.

### 2.7. CyTOF Data Analysis

A total of 36 HCC patients, including 19 VM-subtype and 17 Non-VM-subtype patients, were included for CyTOF analysis. The antibody panel covered 35 immune-related markers. Data preprocessing was performed using the R package cytofkit [16]. To reduce differences in cell numbers across samples, 5000 cells were randomly sampled from each sample and merged for downstream analysis. Marker signal intensities were transformed using the inverse hyperbolic sine transformation. For cell clustering and visualization, the FlowSOM algorithm was first used to construct a self-organizing map, followed by meta-clustering using the PhenoGraph algorithm. A total of 22 cell clusters were identified. The t-SNE algorithm was then used to project high-dimensional CyTOF data into a two-dimensional space for visualization. Marker expression heatmaps were generated to characterize the phenotypic features of each cluster, and cluster abundances were compared between VM and Non-VM subtypes to assess differences in the immune microenvironment.

### 2.8. Mutational Analysis

To characterize somatic mutation differences between VM and Non-VM subtypes in HCC, masked somatic mutation data of the TCGA-LIHC cohort were obtained from the TCGA database. According to the VM molecular classification, 88 VM-subtype cases and 92 Non-VM-subtype cases with available mutation data were included for analysis. MAF-format mutation data were analyzed and visualized using the R package maftools [17]. Oncoplots of the top 20 most frequently mutated genes were generated to compare mutation profiles between VM and Non-VM subtypes. Mutation types and base substitution patterns were also summarized. Tumor mutational burden (TMB) was calculated based on whole-exome sequencing data. For each sample, the total number of somatic mutations was counted and normalized to an estimated exome size of 38 Mb to obtain the TMB score.

### 2.9. Drug Sensitivity Analysis

To identify candidate compounds predicted to potentially reverse the VM-associated transcriptional signature, the top 100 significantly upregulated genes in the VM subtype were extracted and submitted to the CMap database [18] for matching analysis. Compounds with negative connectivity scores were considered potential agents capable of reversing the VM-related expression pattern.

To further assess subtype-associated drug sensitivity patterns, the R package oncoPredict [19] was used to estimate drug half-maximal inhibitory concentration (IC_50_) values for each TCGA-LIHC sample based on drug response data of 198 compounds from the GDSC2 database. Predicted IC_50_ values were compared between VM and Non-VM subtypes, and compounds with significant differences between the two groups were identified as candidate subtype-associated sensitive agents.

### 2.10. Molecular Docking Analysis

Molecular docking analysis was performed to predict the potential binding interactions between ivermectin, the top candidate compound identified by CMap analysis, and the six VM-related core proteins, including HSPA9, TGFA, MAD2L1, PROM1, AGXT, and GCGR. The three-dimensional structure of ivermectin was obtained from the PubChem database and optimized by energy minimization using Chem3D 20.0. The three-dimensional structures of the target proteins were obtained from the UniProt database [20]. Water molecules, ions, and other redundant components were removed using PyMOL 2.6.0, followed by hydrogenation pretreatment.

The processed ligand and protein structures were uploaded to the CB-Dock2 online platform [21] for molecular docking. CB-Dock2 was used to automatically identify potential binding pockets and perform docking simulations based on the AutoDock Vina algorithm. Docking results were evaluated according to binding energy, and the conformation with the lowest binding energy was selected as the predicted optimal binding mode. The docking conformations and key interactions between ivermectin and target proteins were visualized for further interpretation.

### 2.11. Cell Lines and Cell Culture

The murine fibroblast cell line 3T3 and human umbilical vein endothelial cells (HUVECs) were kindly provided by Professor Kathy Qian Luo’s laboratory, Faculty of Health Sciences, University of Macau. The murine hepatocellular carcinoma cell line Hepa1-6 (cat. CRL-1830) was kindly provided by academician Shengcai Lin’s laboratory. The human hepatocellular carcinoma cell line Huh7 and the immortalized rat Schwann cell line RSC96, which is derived from rat sciatic nerve Schwann cells, were obtained from ATCC (American Type Culture Collection; Rockville, MD, USA). Unless otherwise specified, cells were cultured in DMEM supplemented with 10% fetal bovine serum and 1% penicillin-streptomycin (Gibco; Thermo Fisher Scientific, Inc., Waltham, MA, USA), and maintained at 37 °C in a humidified atmosphere containing 5% CO_2_.

### 2.12. Neuronal Signal-Conditioned Medium

Culture supernatants from RSC96 Schwann cells were collected after 48 h of culture and centrifuged at 2000× *g* for 10 min to remove cell debris. The supernatant was then mixed with complete medium at a ratio of 2:1 to prepare conditioned medium.

### 2.13. Co-Culture System

After trypsin digestion and cell counting, tumor cells (Huh7), 3T3 cells, and HUVECs were mixed at a ratio of 5:3:2 for establishment of the co-culture system. The control group was cultured in complete medium, while the experimental group was cultured in neuronal signal-conditioned medium. The co-culture system was monitored by fluorescence microscopy (Evos AUTO2) to evaluate cell-cell interactions and network-like structure formation.

### 2.14. Modified Co-Culture System

Huh7 and 3T3-Clover cells were seeded separately (30,000 cells per chamber) into 2-well silicone culture inserts (ibidi, Gräfelfing, Germany) mounted in 6-well plates, creating a 500 μm cell-free gap between the two cell types. The culture inserts were removed 24 h after cell adherence to allow the two cell types to grow toward each other. Images were captured on day 1 using a fluorescence microscope (Evos AUTO2) and recorded continuously for 7 days.

### 2.15. Establishment of YAP1-Overexpressing Hepa1-6 Cells

To establish a stable YAP1-overexpressing mouse HCC cell line, Hepa1-6 cells were transduced with lentiviral particles carrying the YAP1 overexpression construct. When cell confluence reached 40–50%, lentiviral particles were added at a multiplicity of infection (MOI) of 10 in the presence of Polybrene at a final concentration of 8 μg/mL. At 48 h after transduction, cells were selected with puromycin at 2 μg/mL for 48 h to establish stable Hepa1-6-YAP1OE cells.

### 2.16. Establishment of Mouse Orthotopic Tumor Models

All animal experimental procedures were approved by the Institutional Animal Care and Use Committee (IACUC) of Guangxi Medical University (Approval No. 202601007). Male C57BL/6J mice, aged 8 weeks, were obtained from Guangdong Vital River Laboratory Animal Technology Co., Ltd., Foshan, China. Orthotopic implantation of Hepa1-6-derived mouse HCC cells was performed as previously described [22,23] with minor modifications. Briefly, wild-type Hepa1-6 and Hepa1-6-YAP1OE cells were trypsinized, washed with serum-free DMEM, and resuspended in serum-free DMEM. A total of 1.5 × 10^6^ cells in 40 μL of serum-free DMEM were slowly injected into the left hepatic lobe of the mice (*n* = 7 per group). After injection, the liver surface at the needle insertion site was gently compressed with a sterile cotton swab for approximately 2 min to minimize bleeding and cell leakage, followed by abdominal closure. Mice were maintained for 2 weeks after implantation and then euthanized. Liver tumor tissues were harvested for subsequent histological and molecular analyses. The outcome measurement for this model was primarily based on objective histological assessment; therefore, no blinding was applied during the procedure.

### 2.17. Establishment of Verteporfin Treatment Model

Male C57BL/6J mice, aged 7–8 weeks and weighing 20–25 g, obtained from Guangdong Vital River Laboratory Animal Technology Co., Ltd., China, were used to establish the Verteporfin treatment model. Hepa1-6-YAP1OE cells were prepared as a single-cell suspension in serum-free DMEM, and 40 μL of cell suspension was orthotopically injected into the liver of each mouse as described above. One week after implantation, mice were randomly assigned to the Verteporfin treatment group or vehicle control group using a simple randomization sequence generated by Microsoft Excel, with five mice in each group. This sample size was determined based on previous experience with similar orthotopic models to achieve sufficient statistical power while minimizing animal numbers. Mice in the treatment group received an intraperitoneal (i.p.) injection of Verteporfin at 100 mg/kg in a volume of 0.2 mL once daily for two consecutive days each week. Mice in the control group received an equal volume of vehicle following the same schedule. The primary outcome measure was liver tumor growth, with assessment by histological and molecular quantification. After three weeks of treatment, mice were euthanized, and liver tumor tissues were harvested for subsequent analysis. To minimize selection and allocation bias, the investigator performing the data analysis was blinded to the group allocations.

### 2.18. Periodic Acid-Schiff (PAS) Staining

Paraffin-embedded tissues (human and mouse hepatocellular carcinoma tissues) were serially sectioned at 4 μm thickness. Sections were deparaffinized with xylene and rehydrated through a graded ethanol series, followed by PAS staining. Sections were first oxidized in 0.5% periodic acid solution for 10 min, rinsed thoroughly with distilled water, and then incubated in Schiff reagent (BASO, Cat. No. BA4080A) in the dark for 15–20 min until a light purple-red color appeared. Nuclei were counterstained with hematoxylin for 1–2 min, differentiated in acid ethanol for several seconds, and blued under running tap water. After dehydration through a graded ethanol series and clearing with xylene, sections were mounted with neutral balsam. Images were observed and captured under a light microscope. Glycogen-positive regions appeared purple-red/magenta, and nuclei were stained blue.

### 2.19. CD34 Immunohistochemical Staining

Immunohistochemical staining for CD34 was performed on paraffin-embedded tissue sections from both human hepatocellular carcinoma specimens and C57BL/6 mouse orthotopic hepatic tumor models. Tissue sections (4 μm) were deparaffinized in xylene and rehydrated through a descending graded ethanol series. Heat-induced epitope retrieval (HIER) was performed by immersing sections in boiling EDTA buffer (pH 9.0) for 20 min, followed by natural cooling to room temperature. Endogenous peroxidase activity was quenched by incubation with 3% hydrogen peroxide (H_2_O_2_) for 15 min at room temperature, followed by three 5 min washes in phosphate-buffered saline (PBS, pH 7.4). Non-specific binding was blocked with 5% normal goat serum for 30 min at room temperature. After removing excess blocking solution without washing, sections were incubated with anti-CD34 primary antibody (rabbit monoclonal, Abcam ab81289, diluted 1:2000 in PBS) overnight at 4 °C in a humidified chamber. Following three PBS washes, sections were incubated with horseradish peroxidase (HRP)-conjugated species-matched secondary antibody (goat anti-rabbit IgG, 1:200) at 37 °C for 30min, followed by three additional PBS washes. Immunoreactivity was visualized using 3,3′-diaminobenzidine (DAB) chromogen solution (ZSGB-BIO, ZLI-9018) under microscopic observation for 3 min until brownish-yellow positive signals developed. The reaction was terminated by immersion in distilled water. Sections were counterstained with hematoxylin for 2 min, differentiated in 1% acid alcohol, and blued in running tap water. Finally, sections were dehydrated through graded ethanol concentrations (70%, 80%, 90%, 95%, and 100%), cleared in xylene (two changes, 5 min each), and coverslipped with neutral balsam mounting medium.

### 2.20. Statistical Analysis

Statistical analyses were performed using R 4.4.1 and GraphPad Prism 10.1.2. Between-group comparisons of continuous variables were performed using either Student’s *t*-test or Wilcoxon rank-sum test based on normality and homogeneity of variance tests. Categorical variables were compared using the chi-square test or Fisher’s exact test. The Benjamini-Hochberg method was applied for multiple testing correction to control the false discovery rate. Survival analysis was conducted using the Kaplan-Meier method, and survival differences between distinct VM subtypes were compared using the log-rank test. Correlation analysis was performed using Pearson’s or Spearman’s correlation coefficient. All statistical tests were two-sided, and *p* < 0.05 was considered statistically significant.

## 3. Results

### 3.1. Construction and Validation of a VM-Associated Molecular Subtyping Model in Hepatocellular Carcinoma

**Figure 1:**
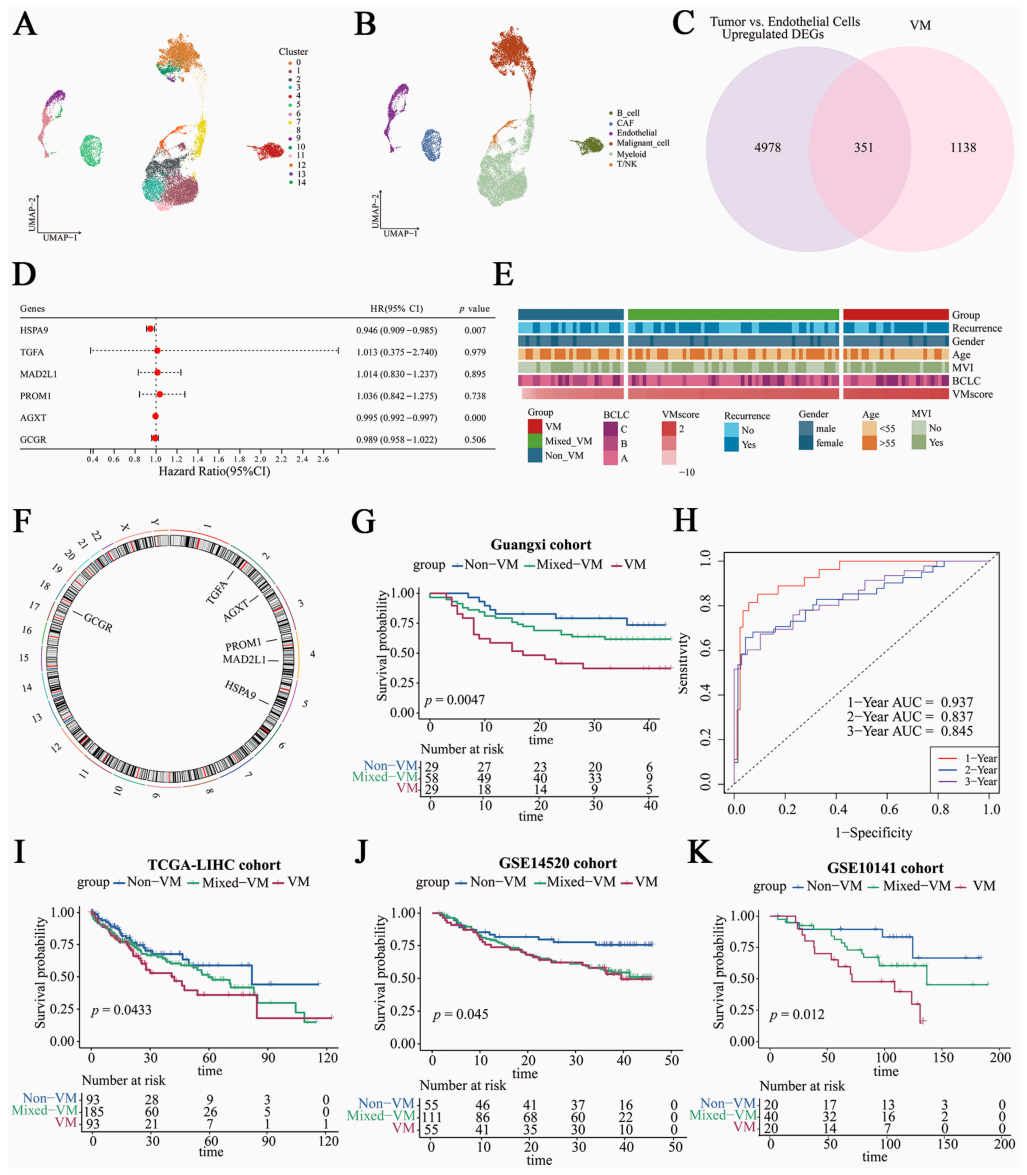
Construction and validation of a VM-associated molecular classification model in HCC. (**A**) UMAP visualization showing 15 cell clusters identified by unsupervised clustering of scRNA-seq data from 10 HCC samples. (**B**) Annotation of 6 major cell types based on the expression of classic marker genes, including B cells, CAFs, endothelial cells, malignant cells, myeloid cells, and T/NK cells. (**C**) Venn diagram showing the intersection between malignant cell-upregulated genes and VM-associated genes. (**D**) Forest plot showing the six-gene VM-associated prognostic signature derived from multivariate Cox regression analysis. (**E**) Heatmap displaying the clinicopathological characteristics of patients stratified into VM, Mixed-VM, and Non-VM subtypes in the Guangxi cohort. (**F**) Circos plot showing the chromosomal localization of the 6 VM-related prognostic genes. (**G**) Kaplan–Meier curves for overall survival (OS) of patients stratified into three VM subtypes based on the prognostic signature in the Guangxi cohort. (**H**) Time-dependent ROC curves evaluating the predictive performance of the VM score for 1-, 2-, and 3-year overall survival. (**I**–**K**) Kaplan–Meier survival analysis validating the OS of the three VM subtypes in the TCGA-LIHC (**I**), GSE14520 (**J**), and GSE10141 (**K**) cohorts, respectively.

Molecular classification provides an important framework for dissecting tumor heterogeneity, improving prognostic stratification, and supporting individualized therapeutic decision-making in HCC. To construct a VM-associated molecular classification model, we first analyzed single-cell RNA-seq data from 10 HCC patients. Unsupervised clustering identified 15 distinct cell clusters (Figure 1A). Based on canonical cell-type marker genes (Figure S1), these clusters were annotated as cancer-associated fibroblasts (CAFs), T/NK cells, malignant epithelial cells, endothelial cells, myeloid cells, and B cells (Figure 1B).

Because VM is considered an endothelium-independent vascular-like structure formed by highly plastic tumor cells, malignant epithelial cells and endothelial cells were extracted for differential expression analysis. A total of 5329 genes were significantly upregulated in malignant epithelial cells. By intersecting these genes with 1489 VM-associated genes collected from public databases and the published literature, we identified 351 HCC-specific VM-related candidate genes (Figure 1C). On this basis, univariate Cox regression, Lasso-Cox regression, and multivariate Cox regression were sequentially employed for stepwise filtering and model optimization. This rigorously culminated in the construction of a VM-based prognostic signature composed of six core genes: *HSPA9*, *TGFA*, *MAD2L1*, *PROM1*, *AGXT*, and *GCGR* (Figure 1D). Protein-level validation using the Human Protein Atlas (HPA) database confirmed that the expression levels of HSPA9, TGFA, MAD2L1, PROM1, and AGXT in HCC tissues were significantly higher than those in adjacent non-tumor tissues (Figure S2).

In the Guangxi HCC cohort, patients were stratified into three subtypes based on their VM risk score: those with scores in the top 25% were defined as the VM subtype, those in the bottom 25% as the Non-VM subtype, and the remainder as the Mixed-VM subtype. To validate the VM subtyping at the histopathological level, PAS/CD34 dual staining was performed on human HCC tissue samples. As shown in Figure S3, VM-subtype tumors exhibited significantly more PAS^+^/CD34^−^ tubular structures characteristic of vasculogenic mimicry compared to Non-VM-subtype tumors, demonstrating strong concordance between the six-gene signature-based molecular classification and the histopathological VM phenotype. Association analysis combining clinicopathological characteristics revealed significant correlations between the VM subtype and advanced BCLC clinical stages, higher rates of tumor recurrence/metastasis, and microvascular invasion (MVI) (Figure 1E), suggesting that this subtype possesses more aggressive biological behavior. The chromosomal locations of the six signature genes were also visualized (Figure 1F). Survival analysis results showed that patients with the VM subtype had the worst prognosis, followed by the Mixed-VM subtype, whereas the Non-VM subtype had the best prognosis, with statistically significant differences among the three groups (Figure 1G). Time-dependent ROC analysis further demonstrated that the VM score had favorable predictive performance for overall survival in the Guangxi cohort (Figure 1H). The aforementioned results were further replicated and validated across three independent external cohorts: GSE10141, GSE14520, and TCGA-LIHC (Figure 1I–K), exhibiting excellent cohort robustness. Furthermore, univariate and multivariate independent prognostic analyses consistently indicated that the VM risk score is an independent prognostic risk factor impacting the overall survival of HCC patients (Figure S4A). Based on independently associated variables, we constructed an integrated nomogram to estimate the 1-, 2-, and 3-year overall survival probabilities of individual patients (Figure S4B). In the nomogram, higher total points were associated with lower predicted survival probabilities. The calibration curves demonstrated generally good agreement between the nomogram-predicted and observed survival probabilities at 1, 2, and 3 years, indicating satisfactory calibration of the integrated prognostic model (Figure S4C). Collectively, these results indicate that the VM-associated molecular classification model can stratify HCC patients into biologically and clinically distinct subgroups and shows stable prognostic performance across multiple cohorts.

### 3.2. Integrated Transcriptomic and Spatial Analyses Reveal Molecular Features Associated with VM Subtypes in HCC

**Figure 2:**
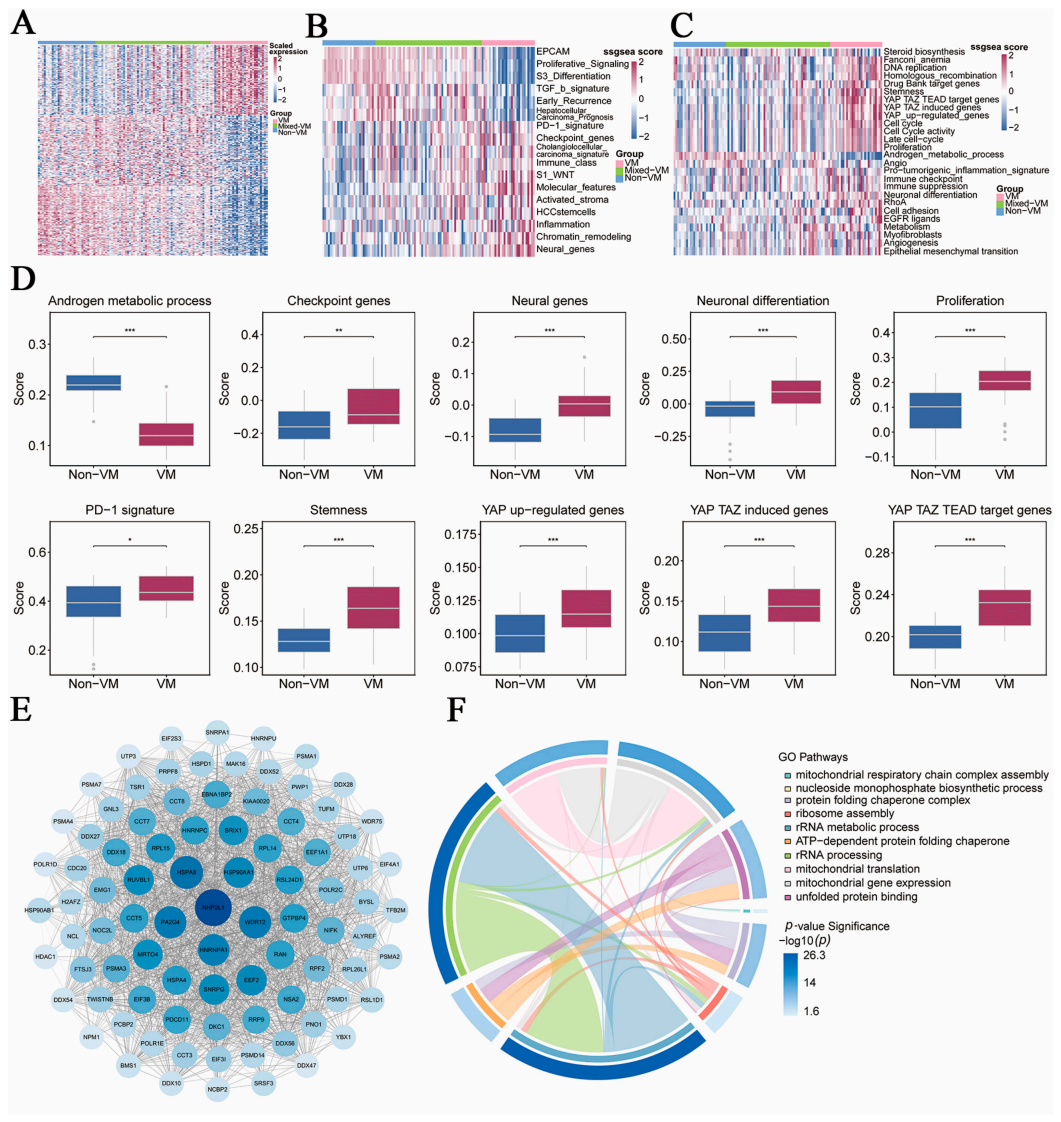
Transcriptomic features and co-expression modules associated with VM subtypes. (**A**) Heatmap showing the top 100 differentially expressed genes among VM, Mixed-VM, and Non-VM subtypes. (**B**,**C**) Heatmaps showing ssGSEA enrichment scores of HCC-related functional gene sets and other biological signatures across the three VM subtypes. (**D**) Box plots comparing representative functional gene set scores between the VM subtype and the Non-VM subtype. (**E**) Protein-protein interaction network of hub genes in the blue module. Node colors represent degree, with darker colors indicating higher degrees. (**F**) GO pathway enrichment analysis of genes in the blue module. Statistical significance is indicated as follows: * *p* < 0.05, ** *p* < 0.01, *** *p* < 0.001.

To characterize the molecular heterogeneity of VM-associated subtypes, we first analyzed bulk transcriptomic profiles across VM, Mixed-VM, and Non-VM subtypes. The three subtypes showed distinct expression patterns, indicating substantial transcriptomic heterogeneity (Figure 2A). Functional gene set analysis further showed that immune checkpoint-related signatures, particularly the PD-1-related signature, and neuronal signaling-related signatures were more enriched in the VM subtype than in the Non-VM subtype. In addition, pathways related to neuronal differentiation, cell proliferation, stemness, and canonical YAP signaling showed higher enrichment in the VM subtype, whereas androgen metabolism-related signatures tended to be lower (Figure 2B–D). These findings suggest that the VM subtype is associated with a distinct transcriptional program involving immune checkpoint activity, neural-related signaling, YAP pathway activation, and stemness/proliferation-related features.

We next analyzed transcription factor (TF) expression patterns among the three subtypes. TF expression profiles also showed subtype-specific clustering, consistent with the overall transcriptomic differences observed among VM, Mixed-VM, and Non-VM subtypes (Figure S5A). Weighted TF enrichment analysis identified ZNF581, POU5F1B, and TP53 as potential regulatory factors associated with subtype-specific transcriptional programs (Figure S5B).

To further identify co-expression modules associated with the VM subtype, WGCNA was performed. Among all modules, the blue module showed the strongest positive correlation with the VM subtype (r = 0.74, *p* = 2 × 10^−10^; Figure S6). GO enrichment analysis showed that genes in this module were mainly involved in protein folding and mitochondrial respiratory chain assembly (Figure 2F), suggesting a potential association with protein homeostasis and mitochondrial function. Protein-protein interaction network analysis further identified HSPA8 and HSP90AA1 as hub genes within this module (Figure 2E). Given the reported involvement of heat shock proteins in tumor progression and angiogenesis-related signaling, these genes may represent potential molecular nodes linking protein homeostasis to VM-associated malignant features.

**Figure 3:**
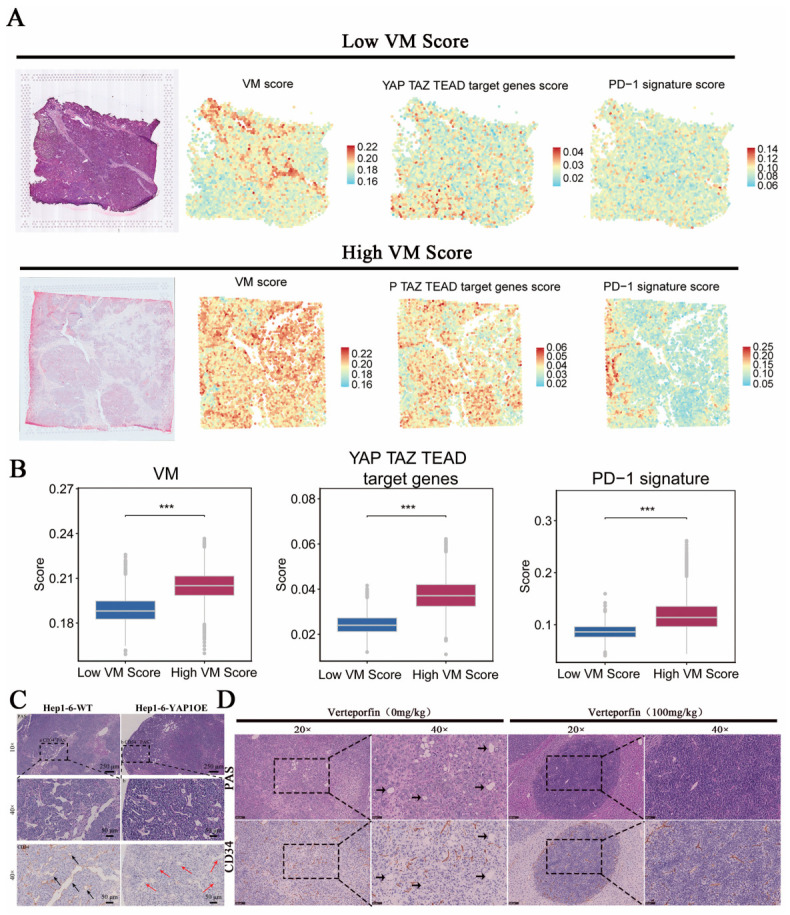
Spatial distribution of VM-associated signatures and histological assessment of VM-like structures. (**A**) Spatial feature plots showing the distribution of VM score, YAP-TAZ-TEAD target gene score, and PD-1 signature score in low-VM-score and high-VM-score HCC tissue sections. (**B**) Box plots comparing the differences in VM score, PD-1 signature score, and YAP-TAZ-TEAD target gene score between patients with high VM scores and low VM scores. (**C**) PAS and CD34 dual staining of orthotopic liver tumors derived from Hepa1-6-WT and Hepa1-6-YAP1OE cells. The red arrow indicates the vascular mimetic lumen (PAS^+^/CD34^−^); the black arrow indicates the traditional endothelial microvessel (PAS^+^/CD34^+^). (**D**) PAS and CD34 dual staining of Hepa1-6-YAP1OE orthotopic tumors treated with vehicle or verteporfin. Arrows indicate representative VM-like structures. Statistical significance is indicated as follows: *** *p* < 0.001.

Spatial transcriptomic analysis was then performed to determine the spatial distribution of VM-associated features in HCC tissues. VM scores were calculated for each spatial spot based on the VM core gene set. High-VM-score regions showed focal enrichment rather than a diffuse distribution pattern. Compared with low-VM-score regions, high-VM-score regions exhibited higher enrichment of YAP-TAZ-TEAD target gene signatures and PD-1-associated signatures (Figure 3A,B).

To further evaluate the association between YAP signaling and VM-like structure formation, we analyzed tumor tissues from orthotopic HCC mouse models established using Hepa1-6-WT and Hepa1-6-YAP1OE cells. PAS and CD34 staining showed that PAS^+^/CD34^−^ VM-like structures were increased in tumors from the YAP1-overexpressing group compared with the control group (Figure 3C). Conversely, treatment with the YAP inhibitor verteporfin reduced the presence of these VM-like structures in Hepa1-6-YAP1OE tumors (Figure 3D). Together, these results support a functional association between YAP pathway activation and VM-like structure formation in HCC.

### 3.3. A Distinct Microenvironment Associated with VM Subtypes

**Figure 4:**
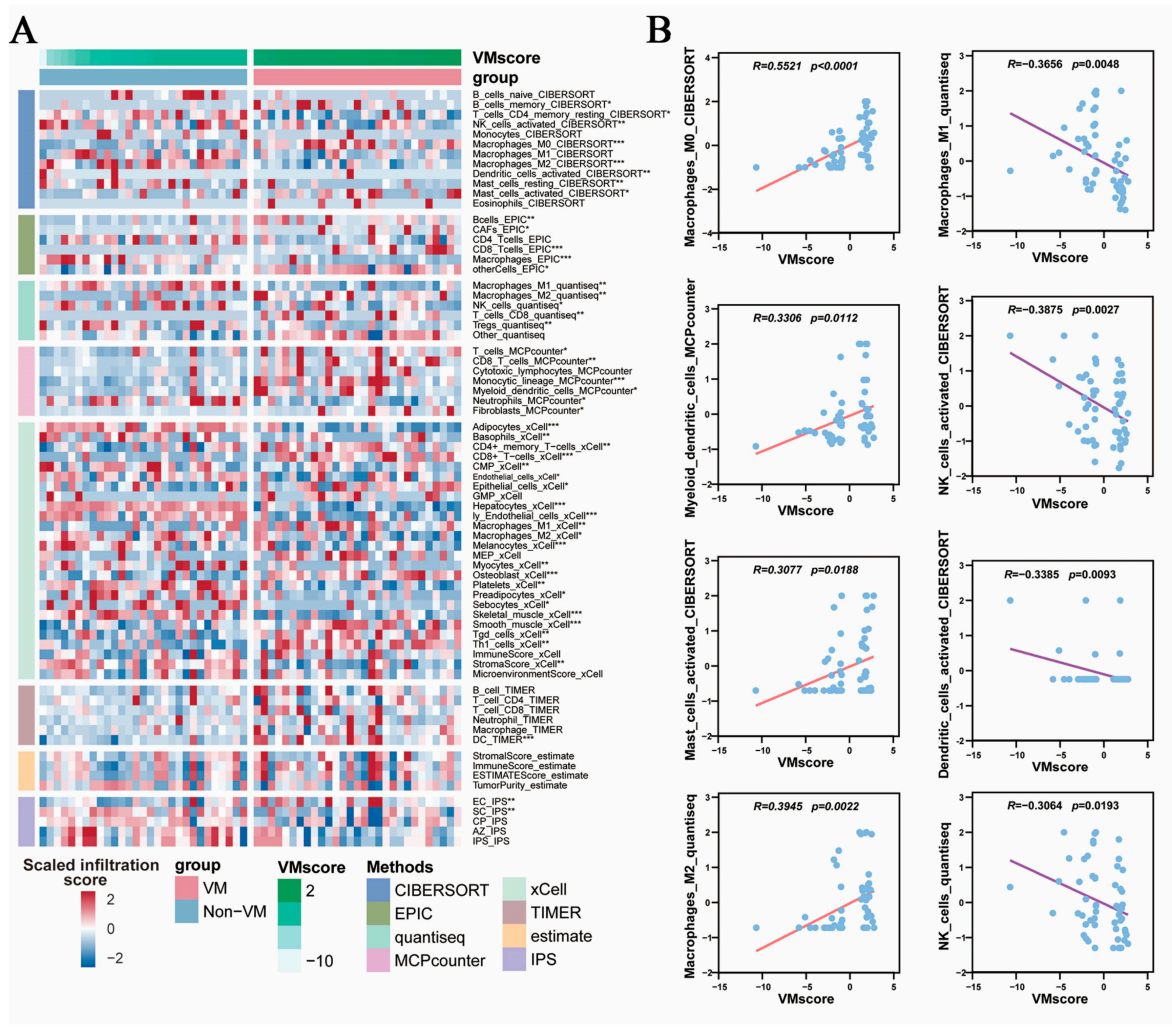
Tumor immune microenvironment composition in VM subtypes. (**A**) Heatmap showing the relative enrichment scores of immune and stromal cell types in VM and Non-VM subtypes, calculated using eight immune infiltration estimation algorithms. (**B**) Correlation analysis between VM score and representative immune/stromal cell populations. The solid lines represent linear regression fits; red lines indicate positive correlations, whereas purple lines indicate negative correlations. Statistical significance is indicated as follows: * *p* < 0.05, ** *p* < 0.01, *** *p* < 0.001.

Given the potential involvement of the tumor microenvironment (TME) in VM-associated tumor progression, we next investigated the immune and stromal features of different VM subtypes. Based on bulk transcriptomic profiles, immune cell infiltration was estimated using eight computational algorithms integrated in the IOBR framework, including CIBERSORT, MCP-counter, and TIMER. The resulting heatmap revealed clear differences in immune and stromal cell enrichment patterns between VM and Non-VM subtypes (Figure 4A). Correlation analysis further showed that the VM score was negatively associated with several immune effector-related cell populations, including resting dendritic cells, M1 macrophages, and NK cells, whereas it was positively associated with M0 macrophages, M2 macrophages, activated mast cells, and myeloid dendritic cells (Figure 4B). These findings suggest that the VM subtype is associated with a remodeled immune microenvironment characterized by altered myeloid cell infiltration and reduced enrichment of selected anti-tumor immune components. To further characterize TME differences between VM and Non-VM subtypes, we applied the xCell algorithm, which provides enrichment estimates for a broad range of immune and stromal cell populations. Consistent with the above findings, xCell analysis revealed distinct cell enrichment profiles between the two subtypes (Figure S7A). Quantitative comparison showed that Th2 cells and monocytes were more enriched in the VM subtype, whereas adipocyte and hepatocyte signatures were higher in the Non-VM subtype (Figure S7B). These results indicate that VM subtypes are accompanied by broad remodeling of immune and stromal components in the HCC microenvironment.

We further analyzed the association between VM score and cytokine expression profiles. Heatmap analysis showed distinct expression patterns of multiple cytokine families, including chemokines, interferons, and interleukins, across samples with different VM scores (Figure S8A). Correlation analysis identified several cytokines positively associated with the VM score, including CSF1, CXCL9, IL1B, TNFSF10, PDGFA, and TGFB3, whereas CCL16, CCL25, and IL27 were negatively associated with the VM score (Figure S8B). Notably, CSF1 is involved in macrophage recruitment and polarization, while IL1B and TGFB3 are closely related to inflammatory signaling, immune regulation, and extracellular matrix remodeling.

**Figure 5:**
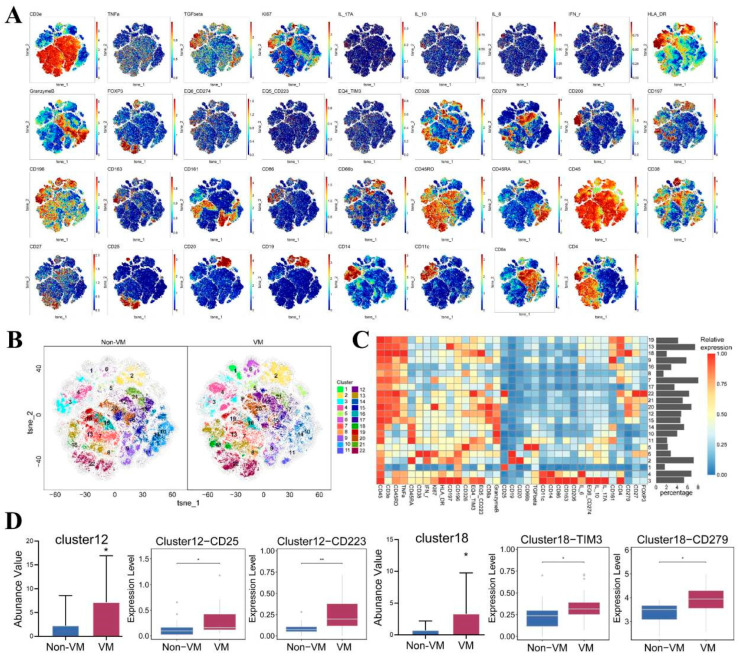
CyTOF analysis reveals distinct immune cell phenotypes between VM and Non-VM subtypes. (**A**) t-SNE feature plots showing the expression distribution of immune cell surface markers across all CyTOF-analyzed single cells. (**B**) t-SNE visualization of CyTOF data from VM and Non-VM tumors, with cells colored by 22 PhenoGraph-defined clusters. (**C**) Heatmap showing the expression patterns of canonical immune markers across the 22 cell clusters. (**D**) Bar plots and box plots comparing the abundance of Cluster 12 and Cluster 18, as well as the expression levels of CD25, CD223, TIM3, and CD279 within these clusters, between VM and Non-VM subtypes. Statistical significance is indicated as follows: * *p* < 0.05, ** *p* < 0.01.

To validate the immune microenvironmental features at single-cell resolution, we performed CyTOF analysis on 172,856 high-quality single cells isolated from 36 HCC tissue specimens. t-SNE visualization based on immune marker expression revealed a heterogeneous immune cell landscape (Figure 5A). Using FlowSOM and PhenoGraph clustering, we identified 22 cell clusters with distinct marker expression profiles (Figure 5B,C). Quantitative analysis showed that two T-cell clusters with inhibitory or exhausted phenotypes, including CD25^+^CD223^+^CD8^+^ T cells (Cluster 12) and TIM3^+^CD279^+^CD4^+^ T cells (Cluster 18), were more abundant in VM-subtype tumors than in Non-VM tumors. In addition, inhibitory checkpoint markers such as CD223 (LAG-3), TIM3, and CD279 (PD-1) showed higher expression in these subsets (Figure 5D). These findings suggest that the VM subtype is associated with enrichment of exhausted or suppressive T-cell populations.

Together, bulk immune deconvolution, cytokine profiling, and CyTOF analysis consistently indicate that the VM subtype is characterized by a distinct immune microenvironment involving myeloid remodeling, cytokine dysregulation, and enrichment of exhausted T-cell phenotypes. These immune features may contribute to the aggressive biological behavior of the VM subtype and provide a rationale for further evaluating immune checkpoint blockade strategies in this patient subgroup.

### 3.4. High-VM Tumor Cells Exhibit Enhanced Microenvironmental Crosstalk and Neural Signal-Conditioned Cell Interactions

**Figure 6:**
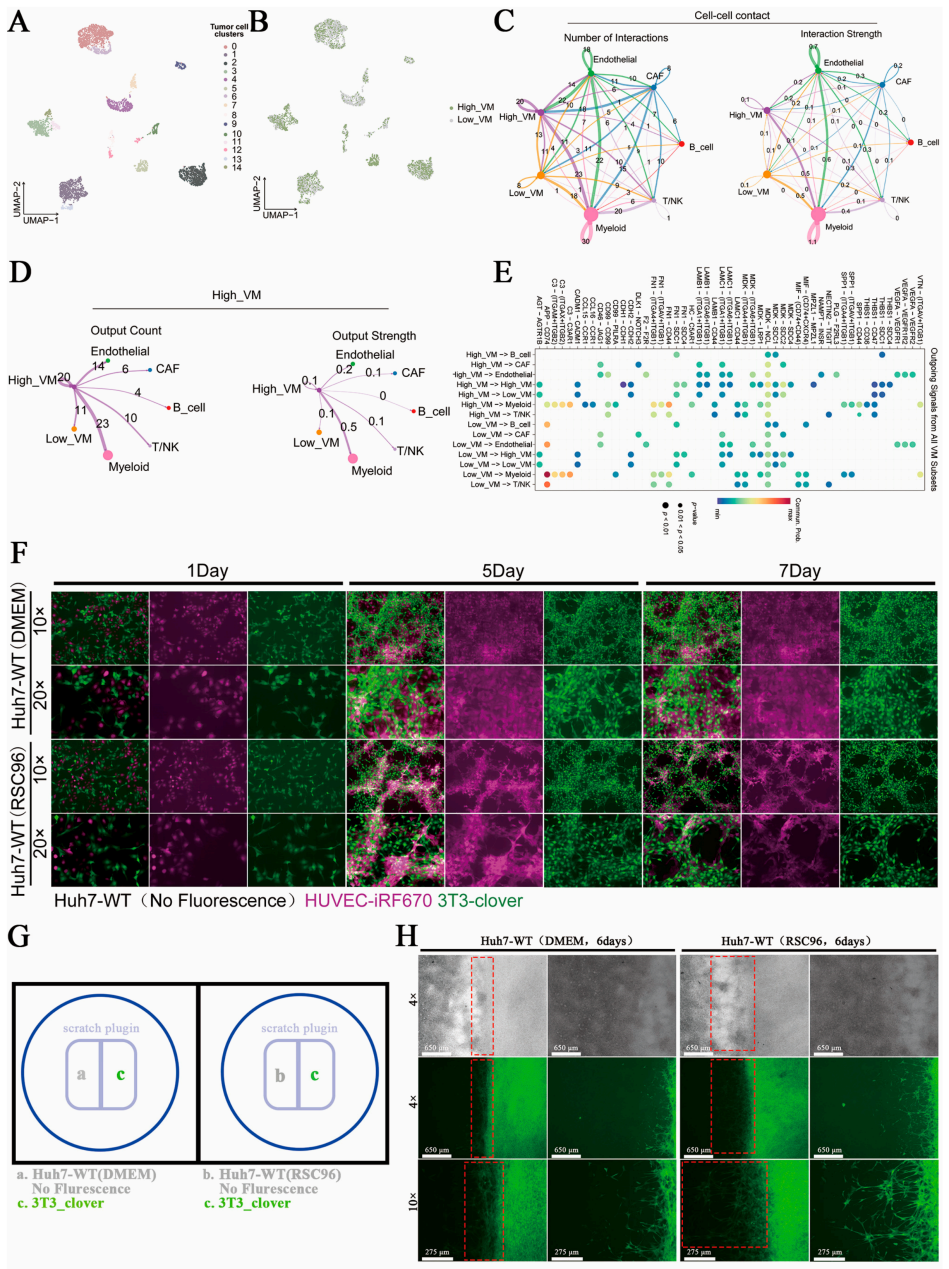
Microenvironmental interaction network of tumor cells with High-VM and Low-VM phenotypes and in vitro validation of neural signal regulation. (**A**) UMAP visualization of 15 tumor cell subclusters. (**B**) UMAP visualization showing tumor cells stratified into High-VM and Low-VM subpopulations according to VM score. (**C**) Cell-cell communication analysis comparing the number and strength of interactions between High-VM or Low-VM tumor cells and major microenvironmental cell populations. (**D**) Outgoing signaling count and strength from the High-VM tumor cell subpopulation to other cell types. (**E**) Bubble plot showing representative differentially enriched ligand-receptor interactions between High-VM and Low-VM tumor cell subpopulations and microenvironmental cells. (**F**) Time-lapse live-cell fluorescence imaging of the multicellular co-culture system composed of Huh7-WT cells, HUVEC-iRFP670 cells, and 3T3-Clover cells cultured under control medium or Schwann cell-conditioned medium. Images were acquired on days 1, 5, and 7. (**G**) Schematic diagram of the modified gap co-culture system used to assess interactions between Huh7 cells and 3T3-Clover fibroblasts. (**H**) Representative live-cell images of Huh7 cells and 3T3-Clover fibroblasts cultured in the modified co-culture system under control medium or Schwann cell-conditioned medium. The red dashed boxes indicate the regions selected for magnified views shown on the right side of the figure.

To investigate tumor cell-microenvironment interactions associated with the VM phenotype, we first performed secondary clustering of malignant epithelial cells from the scRNA-seq dataset and identified 15 tumor cell subclusters (Figure 6A). Based on the VM score derived from the established VM signature, tumor cells were further classified into High-VM and Low-VM subpopulations (Figure 6B). Cell-cell communication analysis showed that High-VM tumor cells exhibited a greater number and higher strength of interactions with key stromal components, particularly endothelial cells and CAFs, compared with Low-VM tumor cells (Figure 6C,D). Signal interaction profiling further indicated that endothelial cell-related signaling interactions were more prominent in the High-VM subpopulation (Figure 6E).

Given the enrichment of neural-related signatures in the VM subtype and the enhanced stromal interactions observed in High-VM tumor cells, we further explored whether Schwann cell-derived signals could influence multicellular interactions relevant to VM-associated multicellular network organization. To this end, we established an in vitro multicellular co-culture system consisting of Huh7 tumor cells, 3T3 fibroblasts, and HUVECs mixed at a ratio of 5:3:2. To enable cell tracking, 3T3 cells were labeled with Clover fluorescence, and HUVECs were labeled with iRFP670 fluorescence. The control group was cultured in complete medium, whereas the experimental group was cultured in Schwann cell-conditioned medium derived from RSC96 cells.

Live-cell fluorescence imaging was then used to monitor multicellular interactions over time. Compared with the control group, cells exposed to Schwann cell-conditioned medium showed more evident network-like organization. HUVECs began to form branch-like structures around day 5, and this pattern became more pronounced on days 6 and 7 (Figure 6F). In addition, HUVECs appeared to extend along fibroblast-rich regions, suggesting that Schwann cell-derived factors may enhance heterotypic interactions among tumor cells, fibroblasts, and endothelial cells in vitro.

We further used a modified gap co-culture system to examine the interaction between Huh7 cells and 3T3-Clover fibroblasts (Figure 6G). Under Schwann cell-conditioned medium treatment, fibroblasts showed more apparent directional migration toward Huh7 cells and displayed elongated or branched morphology compared with the control condition (Figure 6H). These observations suggest that Schwann cell-derived signals may promote tumor cell-fibroblast interactions and induce fibroblast activation-like morphological changes. Together, the cell communication analysis and co-culture experiments support a potential link between neural signal-associated microenvironmental cues and VM-related multicellular organization in HCC.

### 3.5. Transcriptome-Inferred Metabolic Profiling Reveals Distinct Metabolic Reprogramming Across VM Subtypes

**Figure 7:**
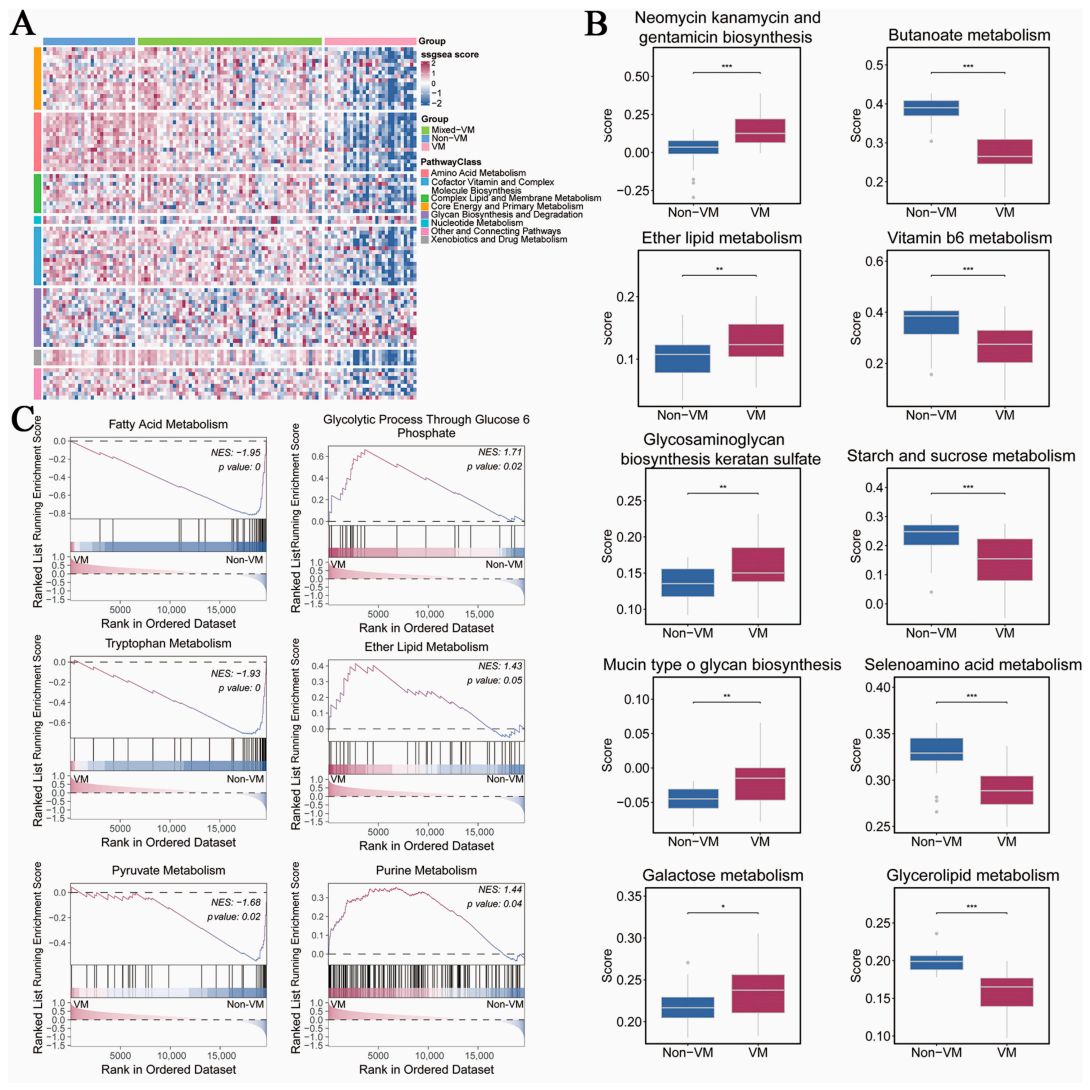
Transcriptome-based metabolic pathway analysis across VM subtypes. (**A**) Heatmap displaying ssGSEA scores of metabolic pathway gene sets across VM, Mixed-VM, and Non-VM subtypes. (**B**) Box plots comparing the enrichment scores of representative metabolic pathway gene sets between VM and Non-VM subtypes. (**C**) Gene Set Enrichment Analysis (GSEA) identifies metabolic pathways differentially enriched between VM and Non-VM subtypes. Statistical significance is indicated as follows: * *p* < 0.05, ** *p* < 0.01, *** *p* < 0.001.

To characterize metabolic heterogeneity across VM subtypes in HCC, we inferred the activity of canonical metabolic pathways using single-sample gene set enrichment analysis (ssGSEA). Distinct and heterogeneous clustering patterns were observed across VM subtypes in the transcriptome-inferred metabolic landscape (Figure 7A). Notably, compared with the VM subtype, the Non-VM subtype displayed higher enrichment scores across a broader range of metabolic pathways. Further subtype-specific pathway analysis revealed divergent metabolic programs between these two subgroups. Compared with the Non-VM subtype, the VM subtype showed higher enrichment in mucin-type O-glycan biosynthesis, glycosaminoglycan biosynthesis-keratan sulfate, galactose metabolism, and ether lipid metabolism. In contrast, pathways related to energy homeostasis and trace element metabolism, including butanoate metabolism, glycerolipid metabolism, selenoamino acid metabolism, starch and sucrose metabolism, and vitamin B6 metabolism, were more strongly enriched in the Non-VM subtype (Figure 7B). Gene set enrichment analysis (GSEA) yielded results consistent with the ssGSEA outcomes. In the VM subtype, purine metabolism, ether lipid metabolism, and the glycolytic process through glucose-6-phosphate were significantly enriched (Figure 7C), suggesting that VM subtype tumors may have increased biosynthetic and glycolytic demands associated with cell proliferation and extracellular matrix remodeling. By contrast, tryptophan metabolism, fatty acid metabolism, and pyruvate metabolism, pathways tightly coupled to oxidative phosphorylation, were more enriched in the Non-VM subtype (Figure 7C). Collectively, these transcriptome-based pathway analyses indicate that the Non-VM subtype is associated with broadly elevated metabolic pathway enrichment, whereas the VM subtype displays a more specific metabolic program characterized by glycolytic activity, ether lipid metabolism, and nucleotide biosynthesis, potentially reflecting metabolic adaptations linked to the VM phenotype in HCC.

### 3.6. Untargeted Metabolomics Identifies Modified Nucleosides and Branched-Chain Keto Acids as VM Subtype Metabolic Features

To further define metabolite-level differences between VM and Non-VM subtypes and identify candidate metabolic biomarkers, we performed untargeted metabolomic profiling on HCC tissue samples classified according to the VM molecular model, including 12 VM-subtype and 10 Non-VM-subtype tumors. In total, 134 high-quality endogenous metabolites were annotated for subsequent comparative analysis.

**Figure 8:**
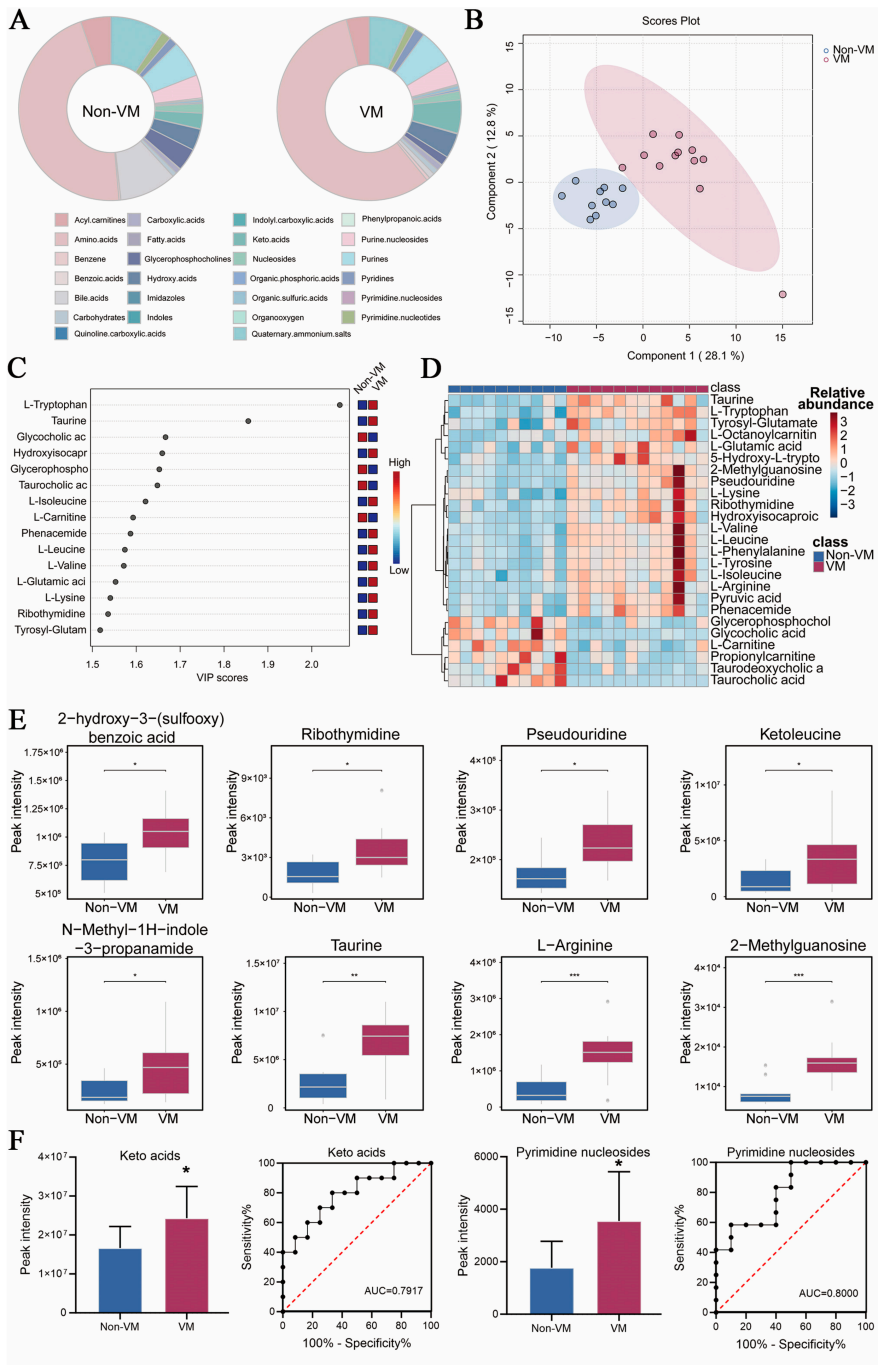
Potential metabolite biomarkers for VM and Non-VM subtypes. (**A**) Relative frequency of metabolites in VM and Non-VM subtypes. (**B**) Partial least squares discriminant analysis (PLS-DA) showing significant differences in metabolites between VM and Non-VM subtypes. (**C**) Variable importance in projection (VIP) scores of altered metabolites. (**D**) Heatmap of the top 25 altered metabolites in VM and Non-VM subtypes. (**E**) Peak intensity of the top 8 significantly differential metabolites in VM and Non-VM subtypes. (**F**) Abundance differences and AUC values of the metabolites keto acids and pyrimidine nucleosides. Statistical significance is indicated as follows: * *p* < 0.05, ** *p* < 0.01, *** *p* < 0.001.

Global metabolomic profiling revealed distinct metabolite composition patterns between the VM and Non-VM subtypes (Figure 8A). To determine whether global metabolic features could distinguish VM from Non-VM subtypes, we performed PLS-DA. The resulting plot demonstrated a distinct separation between the two groups (Figure 8B), indicating differences in metabolite abundance patterns between the two subgroups. To identify key molecular contributors to subtype discrimination, we calculated VIP scores using stringent thresholds of VIP > 1.0 and *p* < 0.05. The top 15 core differential metabolites were further screened based on these criteria (Figure 8C). Heatmap visualization of the top 25 differential metabolites further illustrated distinct expression patterns that accurately discriminate between VM and Non-VM subtypes (Figure 8D). L-arginine, 2-methylguanosine, pseudouridine, taurine, 2-hydroxy-5-sulfobenzoic acid, keto-leucine, *N*-methyl-1H-indole-3-propionamide, and ribothymidine exhibited significantly elevated relative abundance in the VM subtype compared to Non-VM (Figure 8E). Notably, the VM subtype was characterized by increased levels of modified nucleosides, including pseudouridine and ribothymidine, as well as branched-chain amino acid (BCAA)-derived keto acids, such as keto-leucine. The accumulation of modified nucleosides may reflect enhanced RNA turnover, RNA modification, or nucleotide metabolic remodeling in VM-subtype tumors, whereas elevated BCAA-derived keto acids suggest altered BCAA transamination and catabolic activity. These metabolite changes may be associated with the highly plastic and adaptive metabolic state of the VM subtype.

To evaluate their potential clinical utility as discriminative biomarkers, ROC curve analyses were performed, focusing on these core differential metabolites. Results demonstrated that pyrimidine nucleosides and keto acid metabolites not only showed significant elevation in the VM subtype but also achieved high area under the curve (AUC) values, strongly supporting their potential as candidate metabolic biomarkers in tissue for distinguishing VM from Non-VM subtypes of HCC (Figure 8F). In summary, untargeted metabolomics revealed distinct metabolite abundance patterns between VM and Non-VM subtypes. The VM subtype was characterized by elevated modified nucleosides and BCAA-derived keto acids. These metabolites may serve as candidate biomarkers for VM subtype discrimination.

### 3.7. Lipidomic Profiling Identifies Cholesteryl Esters and Sphingolipids as VM-Subtype-Associated Lipid Features

**Figure 9:**
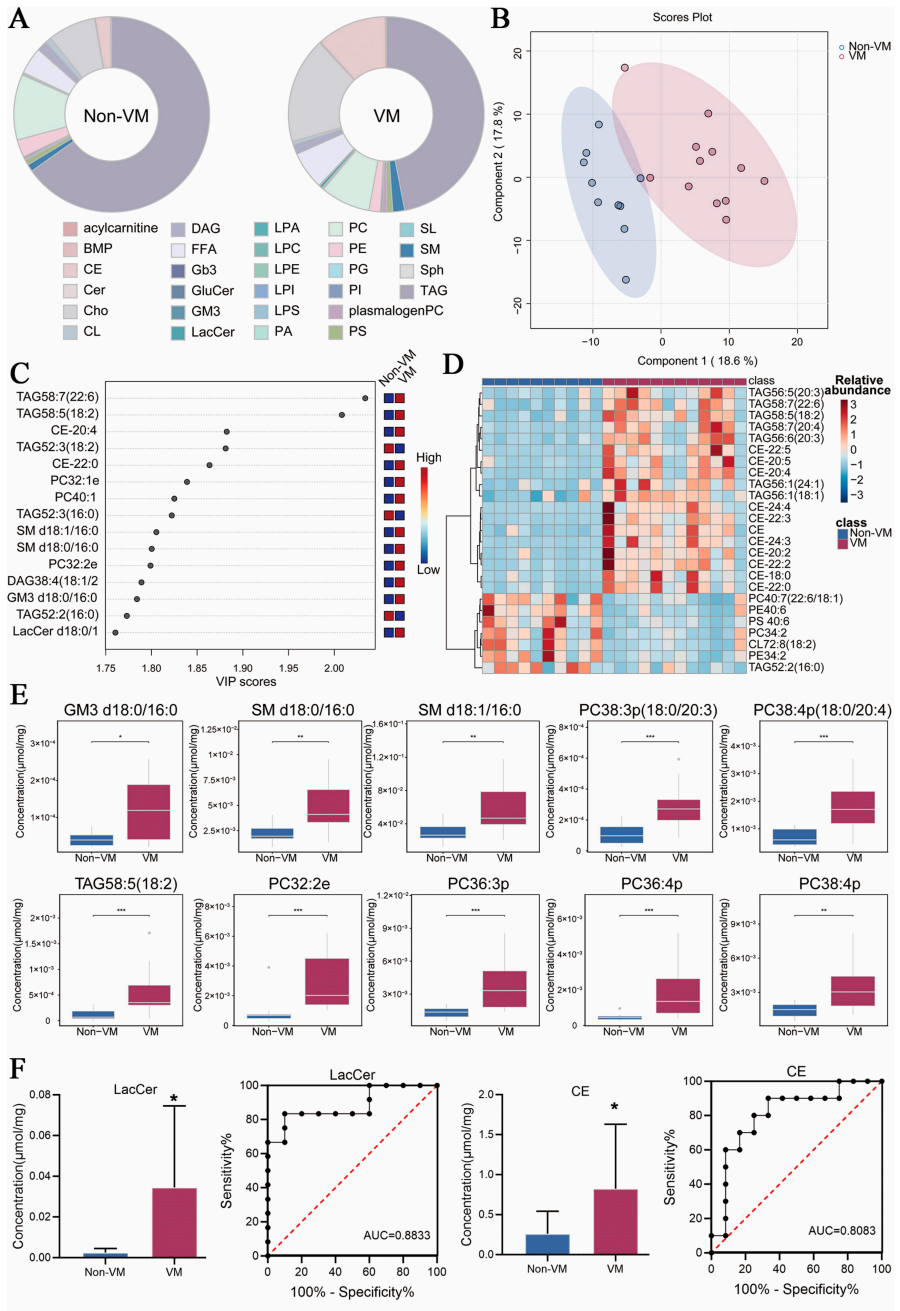
Potential lipid biomarkers for VM and Non-VM subtypes. (**A**) Relative frequency of lipids in VM and Non-VM subtypes. (**B**) PLS-DA showing differentially abundant in lipids between VM and Non-VM subtypes. (**C**) VIP scores of altered lipids. (**D**) Heatmap of the top 25 altered lipids in VM and Non-VM subtypes. (**E**) Peak intensity of the top 10 significantly differential lipid species in VM and Non-VM subtypes. (**F**) Abundance differences and AUC values of the lipids LacCer and CE. Statistical significance is indicated as follows: * *p* < 0.05, ** *p* < 0.01, *** *p* < 0.001.

To characterize lipidomic differences between VM and Non-VM subtypes in HCC and identify candidate lipid biomarkers, we measured the relative abundance of 555 high-quality endogenous lipid species. VM and Non-VM subtypes exhibited different lipid composition patterns, with a relatively clear separation observed between the two groups (Figure 9A,B).

Based on VIP scores, the top 15 lipid species contributing most to subtype discrimination were identified (Figure 9C). Heatmap visualization further showed distinct abundance patterns of these lipid species between VM and Non-VM subtypes (Figure 9D). Quantitative comparison revealed that several lipid species, including GM3 d18:0/16:0, PC33:4p, and SM d18:1/16:0, were significantly increased in the VM subtype compared with the Non-VM subtype (Figure 9E).

Notably, cholesteryl esters (CEs) and lactosylceramide (LacCer)-related lipids were more abundant in the VM subtype (Figure 9F). Increased CE abundance may reflect altered lipid uptake, storage, and cholesterol metabolism, whereas elevated LacCer-related sphingolipids may indicate remodeling of sphingolipid metabolism and membrane-associated signaling. These lipidomic alterations may be associated with the adaptive and invasive biological features of the VM subtype. ROC curve analysis further showed that representative CE and LacCer-related lipids displayed favorable discriminatory performance for distinguishing VM from Non-VM subtypes (Figure 9F).

In summary, lipidomic profiling revealed distinct lipid abundance patterns between VM and Non-VM subtypes in HCC. The VM subtype was characterized by increased CEs and sphingolipid-related species, which may serve as candidate lipid biomarkers for VM subtype discrimination.

### 3.8. Genomic Profiling Reveals Distinct Mutation and Copy Number Variation Landscapes Across VM Subtypes in HCC

**Figure 10:**
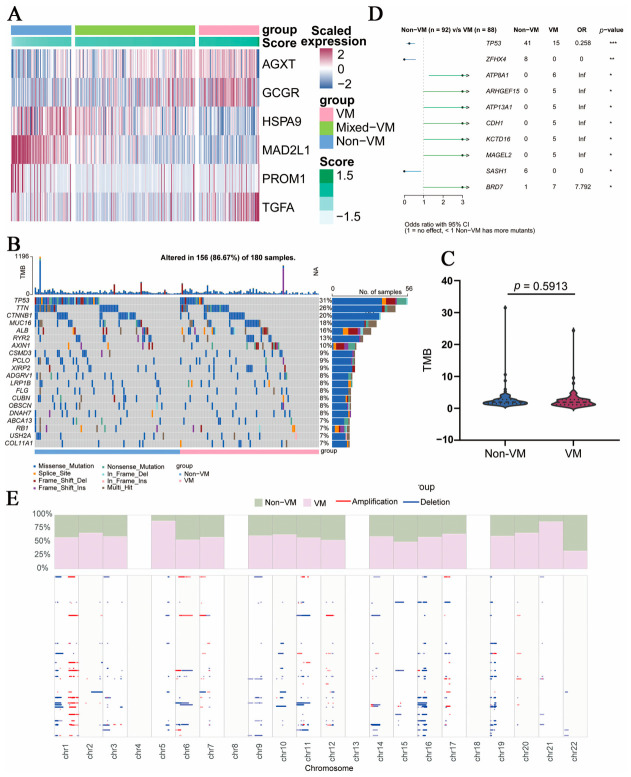
Mutation and copy number variation landscape of VM subtypes in TCGA-LIHC. (**A**) Waterfall plot showing somatic mutation profile differences between VM and Non-VM subtypes. (**B**) Comparison of TMB between VM and Non-VM subtypes. (**C**) Forest plot of differentially mutated genes between VM and Non-VM subtypes. (**D**) Expression heatmap of the six core signature genes across VM, Mixed-VM, and Non-VM subtypes. (**E**) Chromosomal copy number variation (CNV) distribution analysis of VM and Non-VM subtypes. Statistical significance is indicated as follows: * *p* < 0.05, ** *p* < 0.01, *** *p* < 0.001.

To delineate the somatic mutation landscape associated with VM subtypes in HCC, TCGA-LIHC samples were first classified into VM, Mixed-VM, and Non-VM subtypes according to the VM molecular model (Figure 10A). To maximize biological contrast, subsequent mutation analyses focused on the two extreme subtypes, VM and Non-VM, using available whole-exome sequencing data. Among the 180 samples included in the mutation analysis, 156 samples (86.67%) harbored at least one somatic mutation in the top 20 frequently mutated genes displayed in the oncoplot. TP53 was the most frequently mutated gene, with a mutation frequency of 31%, followed by *TTN*, *CTNNB1*, *MUC16*, *ALB*, and *RYR2* (Figure 10B). TMB did not differ significantly between VM and Non-VM subtypes (Figure 10C), suggesting that the VM phenotype was not simply associated with a higher global mutation load. However, differential mutation analysis identified several genes, including *ATP8A1*, *ARHGEF15*, and *ATP13A1*, with higher mutation frequencies in the VM subtype than in the Non-VM subtype (Figure 10D). These findings suggest that VM and Non-VM subtypes may exhibit distinct somatic mutation patterns independent of overall TMB levels.

We further extended our analysis to copy number variations (CNVs). Across the entire cohort, chromosome 1 amplification and chromosome 16 deletion emerged as the most frequent gain and loss events, respectively. Notably, more than half of the samples harboring these CNV events belonged to the VM subtype, indicating a strong subtype-specific enrichment. Furthermore, segmental amplifications and deletions at discrete chromosomal regions also displayed striking enrichment in VM subtype (Figure 10E).

Together, these findings indicate that VM and Non-VM subtypes exhibit distinct genomic alteration patterns, including differences in both somatic mutation profiles and CNV landscapes.

### 3.9. Identification of Candidate Therapeutic Agents for the VM Subtype in HCC

**Figure 11:**
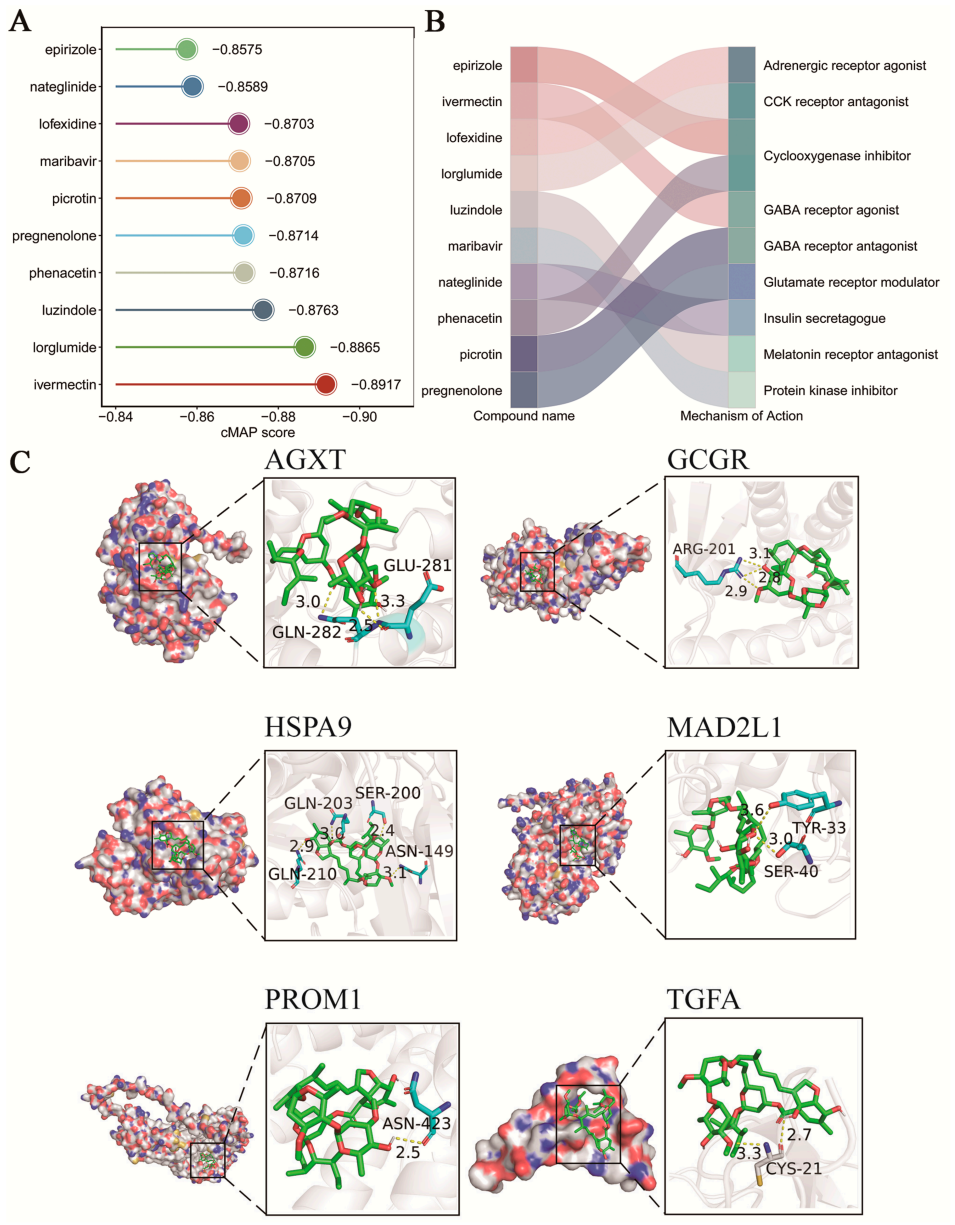
CMap-based candidate drug screening and molecular docking analysis for VM-associated targets. (**A**) CMap analysis showing the top 10 candidate compounds negatively correlated with the VM-related transcriptional signature. (**B**) Sankey diagram showing the predicted mechanisms of action of the top 10 candidate compounds. (**C**) Molecular docking models showing the predicted binding conformations of ivermectin with six VM-related core proteins, including AGXT, MAD2L1, GCGR, TGFA, HSPA9, and PROM1.

Following the detailed characterization of the molecular phenotypes distinguishing VM subtypes in HCC, we next sought to identify potential therapeutic strategies tailored to this high-risk patient population. First, we utilized the CMap database to systematically screen candidate compounds capable of disrupting the VM-specific gene expression signature. Among the top 10 agents predicted to exert selective effects on the VM subtype, ivermectin—a canonical γ-aminobutyric acid (GABA) receptor agonist—showed the strongest negative correlation, with a CMap score of −0.8917 (Figure 11A,B). This robust finding highlights its significant potential to reverse the malignant transcriptomic signature associated with VM. To further elucidate the core molecular basis underlying its inhibitory effects on VM, we conducted a molecular docking analysis. The results demonstrated that ivermectin achieves stable binding conformations with six core VM-related target proteins, including HSPA9, PROM1, and GCGR, exhibiting excellent multi-target binding affinity (Figure 11C, HSPA9: −6.5, PROM1: −10.0, GCGR: −10.8, AGXT: −6.8, MAD2L1: −8.4, TGFA: −8.0 kcal/mol). Together, the CMap prediction and molecular docking results identify ivermectin as a computationally prioritized candidate compound for targeting VM-associated molecular features in HCC. These computational findings provide a rational basis for subsequent functional validation, but the therapeutic potential of ivermectin against VM requires experimental confirmation.

Furthermore, we assessed subtype-associated drug sensitivity using the GDSC2 database. Predicted half-maximal inhibitory concentration (IC_50_) values for 198 compounds were estimated across TCGA-LIHC samples and compared between VM and Non-VM subtypes. A total of 97 compounds showed significant differences in predicted IC_50_ values between the two subtypes *(p* < 0.05), among which 75 were predicted to have lower IC_50_ values in the VM subtype (Supplementary Table S2). These results suggest that VM and Non-VM subtypes may differ in their predicted drug response profiles, and that the VM subtype may be more sensitive to a subset of anticancer agents.

### 3.10. VM Molecular Classification Serves as a Predictive Biomarker for Treatment Response to Sorafenib and TACE Therapy

To evaluate whether the VM molecular classification was associated with treatment benefit in HCC patients receiving standard therapies, we applied the VM classification system to two independent cohorts: the sorafenib-treated cohort and the TACE-treated cohort. Patients in both cohorts were stratified into VM and Non-VM subtypes according to the established VM score, followed by treatment response analysis. Notably, in both the sorafenib cohort (Figure S9A) and the TACE cohort (Figure S9B), patients classified as the Non-VM subtype showed significantly higher treatment response rates than those in the VM subtype. These results suggest that the VM subtype is associated with reduced sensitivity to both sorafenib and TACE therapy, consistent with its aggressive biological features in conventional HCC cohorts.

## 4. Discussion

Tumor heterogeneity is a major challenge in the clinical management of HCC and limits the efficacy of current prognostic and therapeutic strategies. Identifying reliable molecular subtypes and actionable therapeutic targets remains an important goal in HCC translational research. In this study, we focused on VM, a key feature of aggressive HCC, and constructed a VM-based molecular classification system. HCC patients were stratified into three subtypes (VM, Mixed-VM, and Non-VM) with distinct biological behaviors. Survival analysis confirmed that the VM subtype was associated with worse prognosis across multiple independent cohorts. These results support VM as an independent poor prognostic marker and provide a practical tool for risk stratification in HCC.

To clarify the molecular basis of VM-driven HCC progression, we performed multi-omics analyses, including transcriptomics, spatial transcriptomics, CyTOF, metabolomics, and lipidomics. Our results showed that the VM subtype is characterized by enhanced stemness, dedifferentiation, cell cycle dysregulation, and activation of the YAP signaling pathway. These features are consistent with previous reports on VM-positive phenotypes in melanoma and liver cancer, suggesting that VM-related transcriptional programs are closely linked to tumor stemness and dedifferentiation [24]. These findings help explain the high invasiveness and metastatic potential of VM-subtype HCC.

Metabolic reprogramming is a common feature of tumor adaptation and proliferation, and we observed significant subtype-specific metabolic differences in HCC. Metabolomic profiling revealed that the VM subtype is enriched in modified nucleosides, branched-chain keto acids, and specific lipids. These metabolites are involved in energy supply, biosynthesis, and membrane remodeling, which may support the formation and maintenance of VM structures. Consistent with previous studies, these metabolites are also associated with tumor progression and poor prognosis [25,26,27]. Our results suggest that metabolic reprogramming is not only a consequence but also a potential driver of VM phenotypes, providing a basis for targeting metabolic vulnerabilities in VM-subtype HCC.

The tumor immune microenvironment strongly influences therapeutic response and prognosis. Using CyTOF, we found that the VM subtype exhibits an immunosuppressive phenotype, characterized by increased exhausted T cells (e.g., CD25+CD223+CD8+ T cells and TIM3+CD279+CD4+ T cells). These T-cell subsets are closely associated with immune suppression and tumor immune escape [28]. Notably, although the VM subtype is more immunosuppressive, there was no significant difference in TMB compared with the Non-VM subtype. This indicates that immunosuppression in VM-subtype HCC may be driven by intrinsic immune regulation or metabolic remodeling rather than high TMB [29]. These findings highlight the limitations of using TMB alone to assess immune status and support the value of VM-based subtyping for immune evaluation [30].

From a clinical perspective, our VM classification showed predictive value for treatment response. In the sorafenib and TACE cohorts, the Non-VM subtype had significantly better responses than the VM subtype. This suggests that VM may mediate resistance to anti-angiogenic and embolization therapies, consistent with its role in endothelial-independent vascularization. These results indicate that VM subtyping could help guide treatment selection in HCC. In addition, CMap analysis and molecular docking computationally prioritized ivermectin as a candidate compound potentially associated with VM-related molecular features. Ivermectin has been reported to inhibit tumor progression, angiogenesis, and VM formation in HCC and other cancers [31]. However, its effects on HCC growth, VM formation, and related signaling pathways were not experimentally evaluated in this study. Therefore, ivermectin should currently be regarded as a computationally prioritized candidate that requires further in vitro and in vivo validation.

Several limitations should be acknowledged. First, while our six-gene signature shows prognostic value and correlation with VM phenotypes, the direct regulatory roles of these genes in VM formation require functional validation. Although we performed supplementary PAS and CD34 dual staining in the Guangxi HCC cohort for preliminary histological validation, this was limited to a single-center cohort with relatively small sample size. The association between molecularly defined VM subtypes and histologically confirmed VM structures requires validation in larger, multi-center cohorts. Second, although YAP1 overexpression and verteporfin treatment provided functional evidence linking YAP signaling to VM-like tube formation, gene-specific perturbation and rescue experiments are needed to establish causal relationships among the six-gene signature, YAP activity, and VM development. Third, some single-cell, spatial transcriptomic, metabolomic, and lipidomic analyses were performed on relatively small sample sizes, which may limit generalizability. Additionally, the therapeutic response analyses were retrospective and may be subject to selection bias, treatment heterogeneity, and confounding factors. Prospective studies are warranted to validate the predictive value of VM subtypes for treatment response. Fourth, ivermectin was identified through CMap analysis and molecular docking as a candidate compound but has not been functionally validated. Its effects on HCC growth, VM formation, and associated signaling pathways require experimental confirmation in vitro and in vivo. Finally, while our findings were validated across multiple independent cohorts, prospective clinical validation and mechanistic studies in diverse populations will be valuable for clinical translation.

In summary, this study established a VM-based molecular classification system for HCC and systematically characterized the genomic, immune, metabolic, and therapeutic features of VM subtypes. Our findings provide a useful framework for prognostic stratification and personalized therapy in HCC. Future studies will focus on exploring key regulatory mechanisms of VM and developing targeted therapeutic strategies for VM-subtype HCC, with the aim of improving clinical outcomes for high-risk patients.

## 5. Conclusions

In this study, we established and validated a novel molecular classification system for hepatocellular carcinoma based on VM-associated gene signatures. By integrating single-cell transcriptomics, bulk RNA-seq, and multi-omics data from both clinical cohorts and experimental models, we stratified HCC patients into three distinct subtypes, namely the VM subtype, the Mixed-VM subtype, and the Non-VM subtype. These three subtypes exhibited significantly different prognostic outcomes, transcriptional programs, immune microenvironmental features, metabolic and lipidomic profiles, and genomic alteration patterns. The VM subtype, characterized by YAP pathway activation, stemness, immunosuppressive T-cell exhaustion, and specific metabolite alterations including modified nucleosides and cholesteryl esters, was associated with poor prognosis and reduced response to sorafenib and transarterial chemoembolization.

Our findings provide a robust framework for prognostic stratification and offer insights into the biological drivers of VM-aggressive HCC. The identification of ivermectin through CMap analysis and molecular docking highlights it as a computationally prioritized candidate for targeting the VM subtype. This VM-based classification system provides a practical framework for risk stratification and may inform subtype-directed therapeutic strategies in hepatocellular carcinoma.

## Data Availability

Publicly available datasets analyzed in this study are accessible from the repositories and accession numbers specified in the manuscript. Data generated from the independent clinical cohort, CyTOF analysis, metabolomic and lipidomic profiling, histological analyses, and experimental models are available from the corresponding author upon reasonable request.

## Supplementary Materials

The following supporting information can be downloaded at: https://www.mdpi.com/article/10.3390/cancers18152482/s1, Figure S1: Annotation of 15 cell clusters from single-cell RNA-seq data of 10 HCC patients. Figure S2: Immunohistochemical validation of the expression characteristics of 5 core proteins in the VM prognostic model in hepatocellular carcinoma tissues. Figure S3 Representative pathological images of vascular structures in VM, Mixed-VM, and Non-VM subtypes. Figure S4. Independent Prognostic Value of VM score and Nomogram Development. Figure S5: Transcription factor expression profiles and core regulatory factor analysis of the three VM subtypes. Figure S6: Key co-expression modules highly associated with VM subtypes identified by WGCNA. Figure S7: xCell-based cell enrichment analysis of the tumor microenvironment in VM and Non-VM subtypes. Figure S8: Correlation analysis between VM subtypes and cytokine expression profiles. Figure S9: Treatment response rates according to VM status in sorafenib and TACE cohorts. Table S1: VM-associated genes. Table S2: Compounds with significantly different predicted IC50 values between the two subtypes.
