# Supplementary material for: Multi-Omics Identification of Vasculogenic Mimicry-Associated Molecular Subtypes in Hepatocellular Carcinoma for Prognostic Stratification and Therapeutic Response Prediction
Source: Cancers (Basel). 2026 Aug 2;18(15):2482. doi: 10.3390/cancers18152482 (PMC13464967; doi:10.3390/cancers18152482)
Supplement: Supplementary file 1 [file cancers-18-02482-s001.zip › supplementary Figure.pdf]

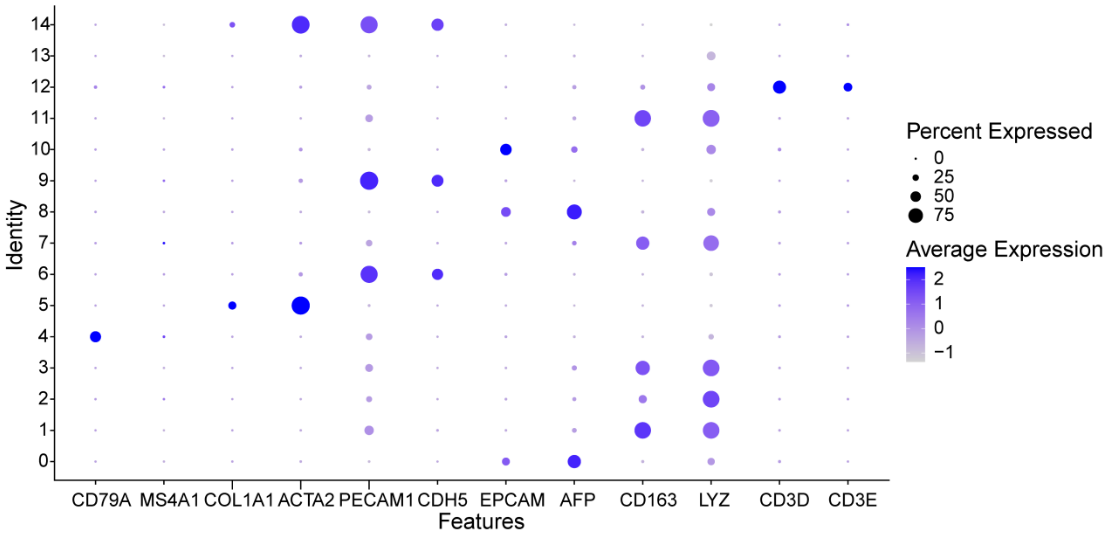

Figure S1. Annotation of 15 cell clusters from single-cell RNA-seq data of 10 HCC patients. Heatmap illustrating canonical marker gene expression patterns.

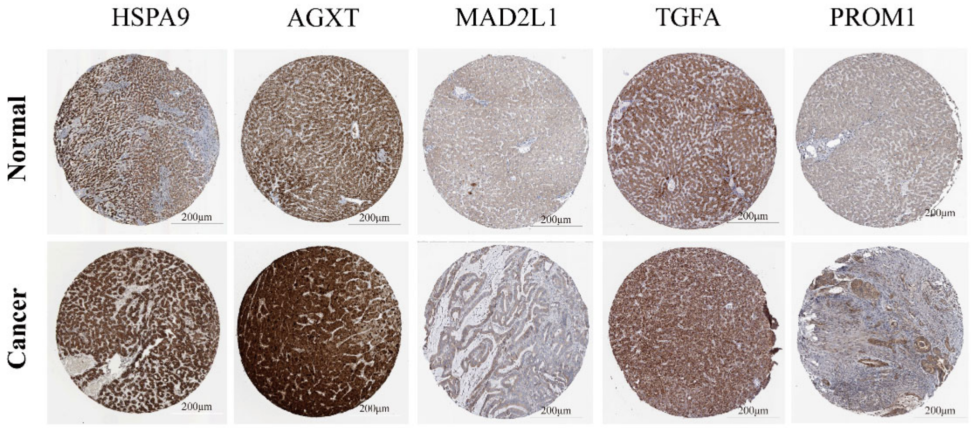

Figure S2. Immunohistochemical validation of the expression characteristics of 5 core proteins in the VM prognostic model in hepatocellular carcinoma tissues.

Immunohistochemical staining showed that the expression levels of HSPA9, AGXT, MAD2L1, TGFA, and PROM1 were significantly upregulated in hepatocellular carcinoma tumor tissues compared with normal liver tissues. (Scale bar: 200 μm)

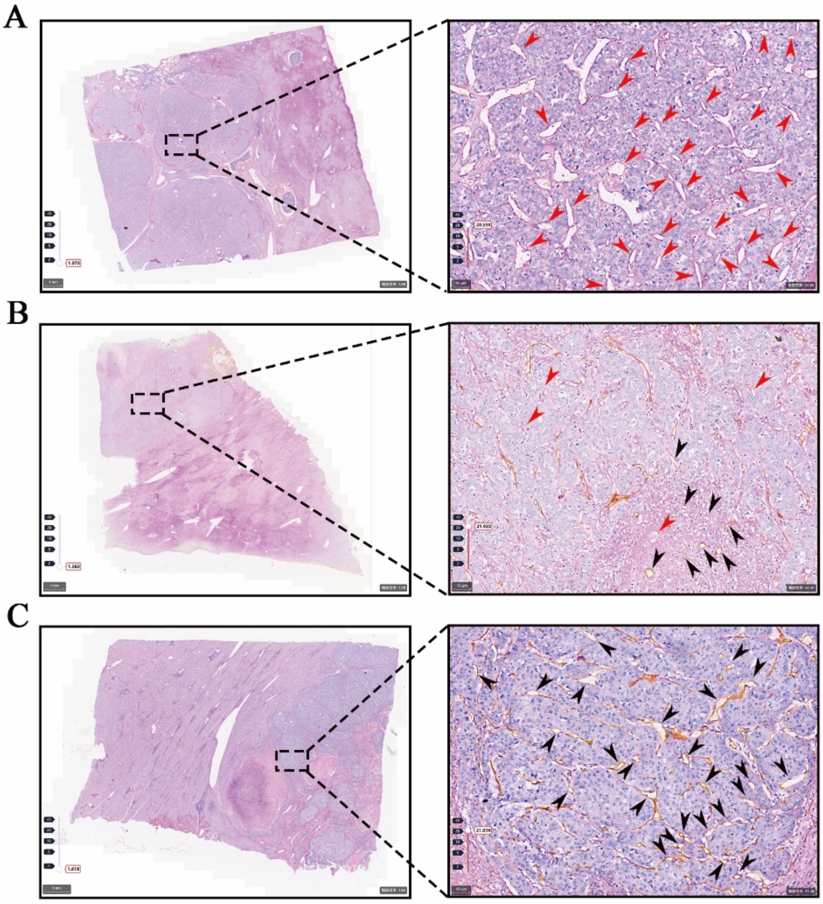

Figure S3. Representative pathological images of vascular structures in VM, Mixed-VM, and Non-VM subtypes. (A) Representative image of PAS/CD34 dual staining in VM subtype tumor tissue. (B)Representative image of PAS/CD34 dual staining in Mixed-VM subtype tumor tissue. (C)Representative image of PAS/CD34 dual staining in Non-VM subtype tumor tissue. The whole slide scan is on the left, and the area within the dotted box is shown enlarged on the right. The red arrow indicates the vascular mimetic lumen ($PAS^+/CD34^-$); the black arrow indicates the traditional endothelial microvessel ($PAS^+/CD34^+$). The left scale bar = 1 mm; the enlarged right scale bar = 50 μm.

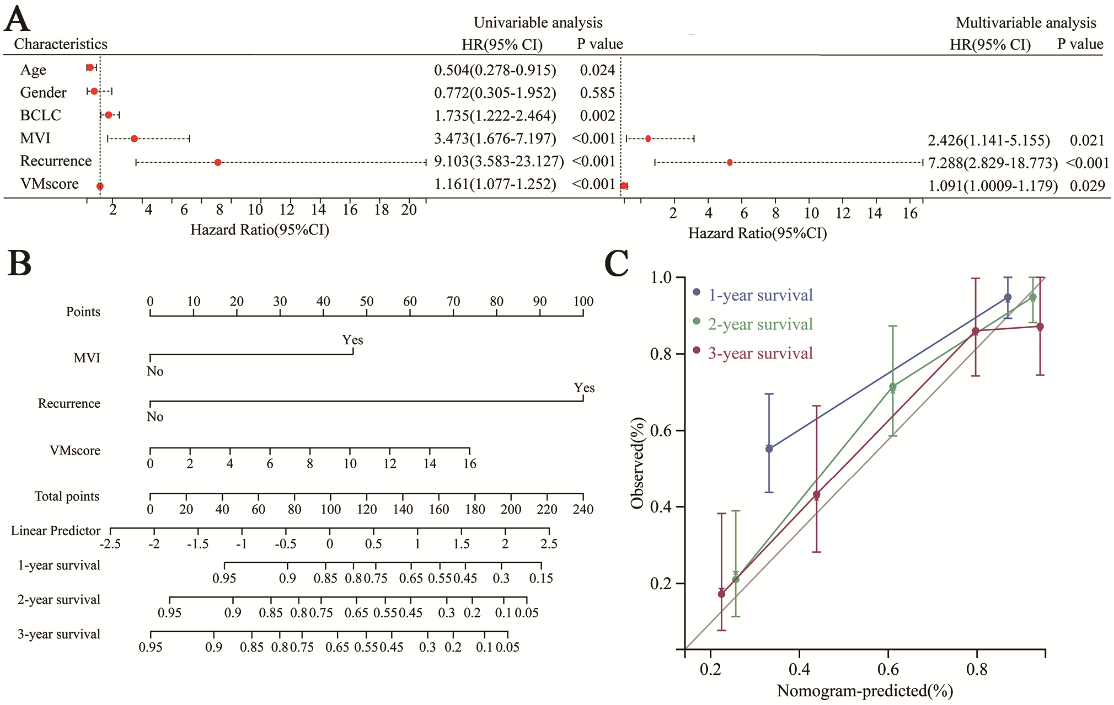

Figure S4. Independent Prognostic Value of VM Score and Nomogram Development. (A) Both univariate and multivariate analyses confirmed that the VM score is an independent prognostic factor. (B) Nomogram incorporating MVI, recurrence status, and VMscore for predicting 1-, 2-, and 3-year survival probabilities. Higher total points indicate a lower predicted survival probability. (C) Calibration curves for the prediction of 1-, 2-, and 3-year survival. The 45° diagonal line represents perfect agreement between the predicted and observed survival probabilities.

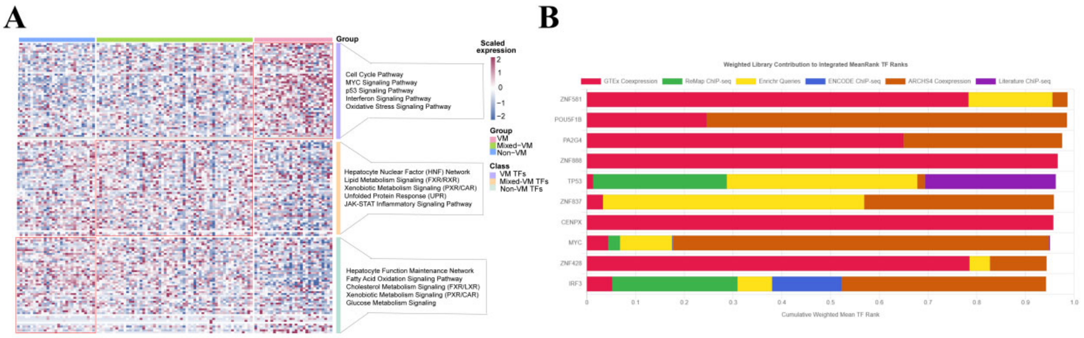

Figure S5. Transcription factor expression profiles and core regulatory factor analysis of the three VM subtypes. (A) Heatmap of transcription factor expression profiles in VM, Mixed-VM, and Non-VM subtypes. (B) Weighted enrichment analysis showing the core transcription factors driving transcriptomic differences in VM subtypes and their regulatory contribution.

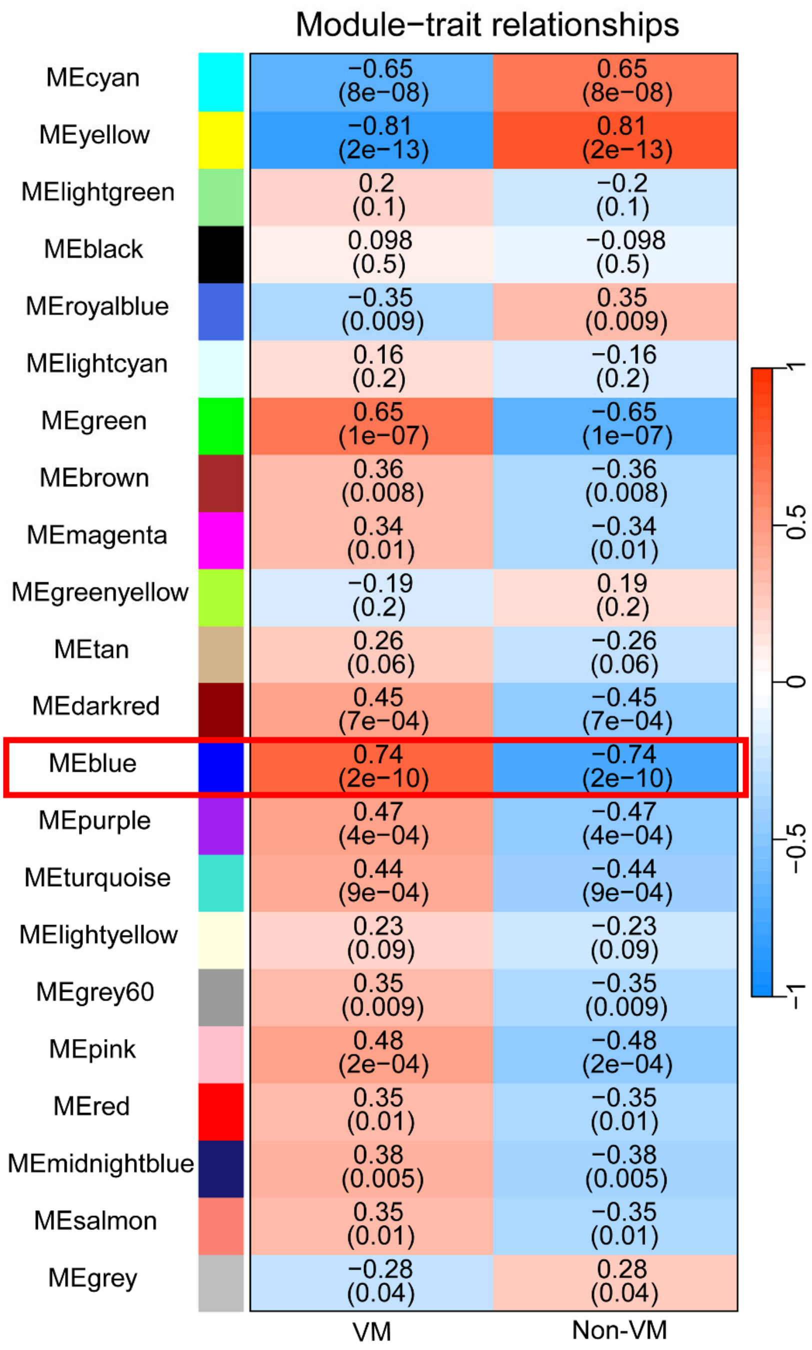

Figure S6. Key co-expression modules highly associated with VM subtypes identified by WGCNA.

Heatmap of module-trait relationships showing the correlation coefficients and corresponding P values of key co-expression modules with VM and Non-VM subtypes.

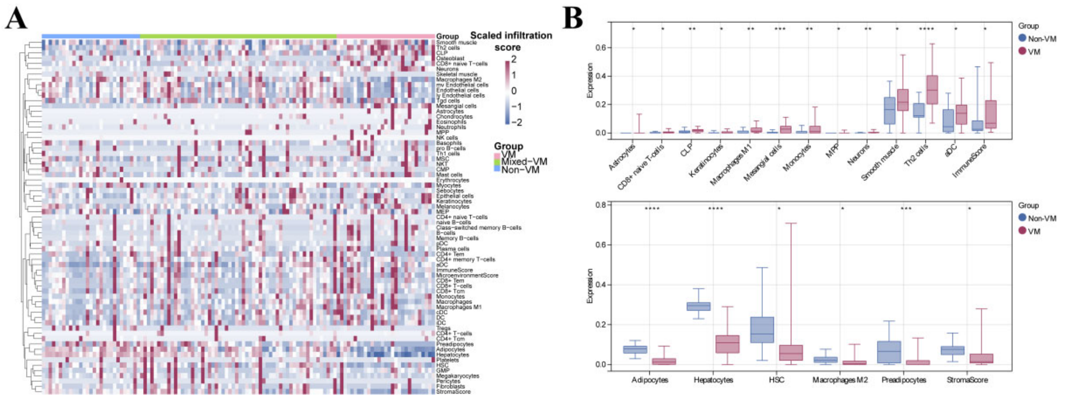

Figure S7. xCell-based cell enrichment analysis of the tumor microenvironment in VM and Non-VM subtypes. (A) Heatmap of immune and stromal cell enrichment profiles in VM, Mixed-VM, and Non-VM subtypes. (B) Box plots showing quantitative analysis of differentially enriched immune and stromal cell components between VM and Non-VM subtypes.

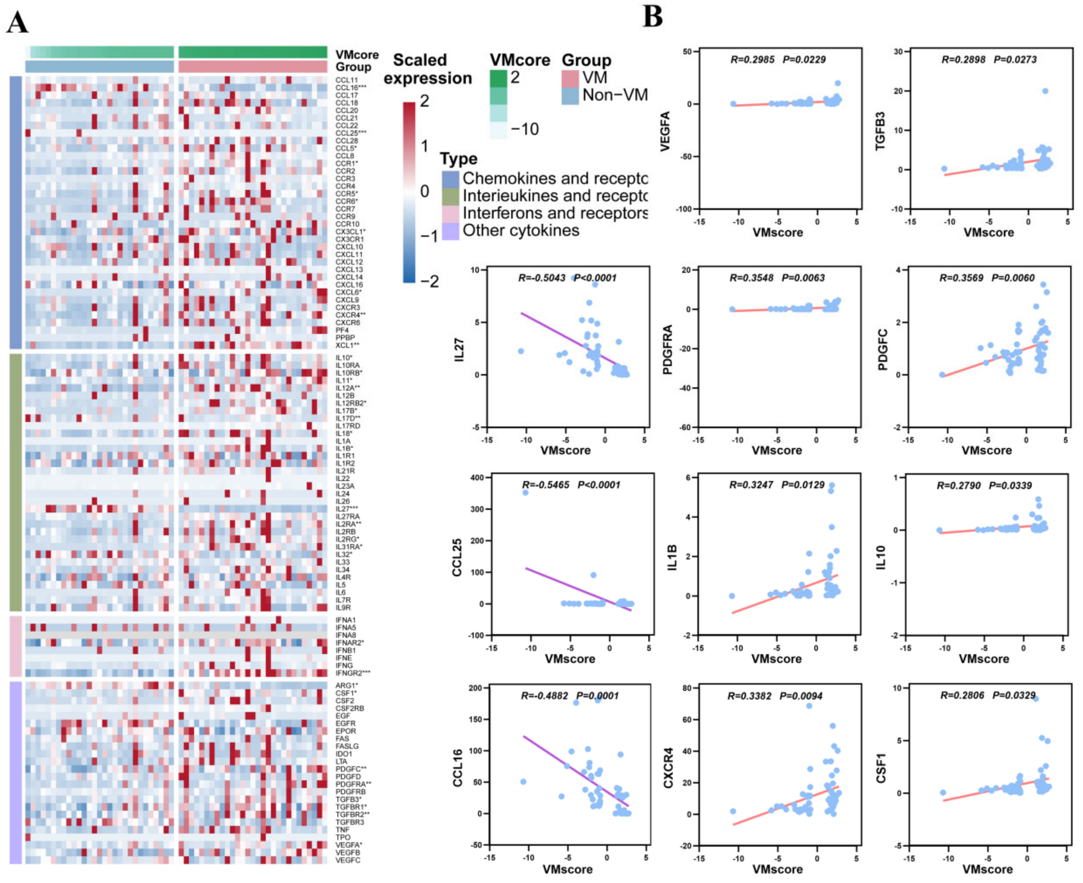

Figure S8. Correlation analysis between VM subtypes and cytokine expression profiles. (A) Heatmap of the expression profiles of various cytokines, including chemokines, interleukins, and interferons, in VM and Non-VM subtypes. (B) Scatter plots showing the correlation between VM scores and the expression of key cytokines.

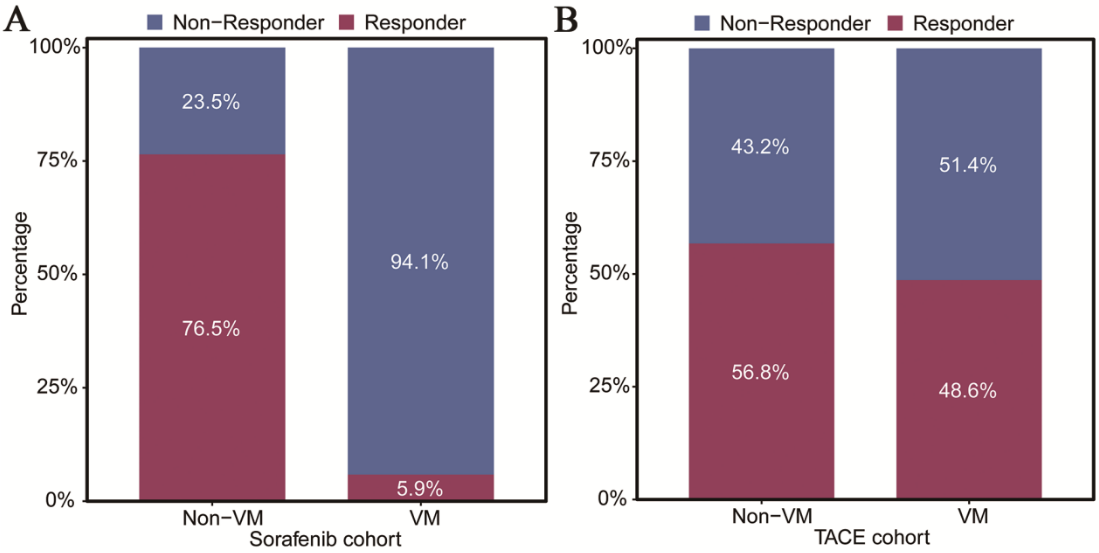

Figure S9. Treatment response rates according to VM subtype in sorafenib and TACE cohorts. (A-B) Stacked bar charts comparing responder and non-responder proportions between Non-VM and VM subgroups in the sorafenib-treated cohort (A) and the TACE-treated cohort (B).
